# The Football Association Injury and Illness Surveillance Programme: The Incidence, Burden, and Severity of Injuries in English Men's Domestic Football

**DOI:** 10.1111/sms.70378

**Published:** 2026-10-03

**Authors:** Bradley Sprouse, Avinash Chandran, Philip Hennis, John Morris, Simon Cooper, Charlotte Cowie, Subhashis Basu, Ian Varley

**Affiliations:** ^1^ Nottingham Trent University School of Science and Technology Nottingham UK; ^2^ Datalys Center for Sports Injury Research and Prevention Indianapolis Indiana USA; ^3^ The Football Association Burton‐Upon‐Trent UK; ^4^ The English Football League Preston UK

**Keywords:** epidemiology, hamstring injury, professional football, time‐loss injuries, time‐trends

## Abstract

Due to the lack of large‐scale research investigating the epidemiology of injury in English men's professional football, the present study aimed to examine the incidence rate, severity, and burden of injury in English men's domestic football. Injury surveillance data was prospectively collected and retrospectively analyzed. Time‐loss injuries, and match and training exposure, were collected by club medical staff across 12 seasons (2013–2014 to 2024–2025) from English men's domestic clubs playing in the English Premier League and English Football League (EFL—Championship, League One, League Two) (416 team seasons). A bootstrapped negative binomial approach was adopted to calculate incidence rate and burden estimates, temporal analysis and incidence rate (IRR) and burden ratios (IBR), all with accompanying 95% CI. 15 986 time‐loss injuries were recorded, resulting in 498 108 days absent over 2 286 030 exposure hours. Injury incidence rate was substantially higher in matches than training (24.9 vs. 3.6 injuries/1000 h; IRR: 7.0 [6.6–7.4]), as was injury burden (835.5 vs. 104.1 days absent/1000 h; IBR: 8.0 [7.5–8.7]). Match injury incidence rate remained stable across the study period, training injury incidence rate decreased by 4% per season (*p* < 0.001), but match injury burden increased by 5% (*p* < 0.001). Muscle injury incidence rate increased by 3% per season in matches (*p* = 0.004), but decreased by 3% in training (*p* = 0.028). Muscle injury burden increased in both matches (10%, *p* < 0.001) and training (4%, *p* = 0.006). Ligament injury incidence rate decreased by 5% per season in training (*p* < 0.001). Hamstring injuries were the most common diagnosis (1.33 injuries/1000 h) and contributed the greatest overall burden (40.37 days absent/1000 h). Over 12 seasons in English men's professional football, match‐play consistently accounts for a greater injury incidence rate and burden than training. While match injury incidence rate has remained stable, burden has increased, specifically for muscle‐related injuries.

## 1 Introduction

Injury surveillance is a cornerstone of sports medicine practice in professional football due to its direct implications for player welfare, team performance, and financial sustainability [1, 2]. Injuries reduce player availability, disrupt tactical continuity, and are associated with poorer competitive outcomes, with lower injury incidence rate and burden linked to improved league success [2, 3, 4]. Beyond performance consequences, injury epidemiology provides the foundation for evidence‐informed prevention strategies, resource allocation, and medical decision‐making, consistent with established models of injury prevention [5]. Systematic surveillance allows practitioners to quantify injury incidence rate, time loss, and burden, and to evaluate the effectiveness of rule changes or preventative interventions over time. In an elite environment, characterized by increasingly high‐intensity match demands [6] and fixture density [7, 8, 9], continuous and methodologically robust surveillance is essential.

Despite the recognized importance of surveillance, much of the existing literature in professional football is constrained by short observation periods and small team samples. Many epidemiological studies span three seasons or fewer [10, 11] and frequently include a limited number of clubs [12, 13]. There is also a scarcity of injury burden analyses, despite being considered a more crucial concept than simply focusing on injury incidence rate [14]. Small samples increase susceptibility to random seasonal variation, reduce generalisability, and limit the ability to detect meaningful longitudinal trends for both incidence rate and burden. Furthermore, methodological inconsistencies, such as variation in injury definitions, time‐loss criteria, and exposure recording, impair comparability across studies [15]. Within English men's professional football, specifically, contemporary research remains limited to single‐season analyses or small cohorts of teams [16], constraining the development of robust benchmark values for applied practice. Although larger‐scale epidemiological investigations have previously been conducted within this population [1, 17], these studies were undertaken over 20 years ago and therefore do not reflect the modern high‐intensity demands and structural changes of the contemporary game.

Large‐scale longitudinal surveillance programmes, most notably the Elite Club Injury Study (ECIS) and datasets presented in Major League Soccer (MLS), have demonstrated the value of sustained multi‐team, multi‐season datasets in detecting temporal trends, quantifying injury severity, and establishing stable benchmark values within elite football overall and for specific injury diagnoses [18, 19, 20, 21]. Such designs provide the statistical power and ecological validity necessary to distinguish meaningful shifts in injury patterns from seasonal variability, which is lacking in the English domestic game. Therefore, the aim of the present study was to examine the incidence rate, burden, and severity of injury during matches and training in English men's professional domestic football across 12 seasons (2013–2025), determining whether there have been any seasonal trends in injury incidence rate and burden over this time period.

## 2 Materials and Methods

### 2.1 Design

The present study conducted a retrospective analysis of prospectively collected surveillance data as part of the *English Football Association (FA) Injury and Illness Surveillance Programme (IISP)*. The present study's research design is outlined below, following the same research design as previous studies examining different subsets of data [22, 23]. Ethical approval for the retrospective analysis of the data was granted by the Nottingham Trent University Non‐Invasive Ethical Review Committee (application number: 116 V2).

### 2.2 Participants

The present study was based on data from English men's professional domestic football collected across 12 seasons (2013–2025) from clubs playing in the English Premier League (2016–2017 to 2020–2021), Championship (2013–2014 to 2024–2025), League One (2016–2017 to 2024–2025), and League Two (2016–2017 to 2024–2025). The sharing of data was voluntary up until the 2021–2022 season and then mandated thereafter. The sample size was convenience sampled, with the number of players determined by the size of the squads of the clubs that consented to involvement in the study each season. There were 416 individual team seasons, with an average of 3, 16, 12, and 11 teams per season in the Premier League, Championship, League One, and League Two, respectively. The sample characteristics from the 12 seasons of data collection are described in Table 1, with data stratified by league described in Table S1. There was a total of 89 unique clubs who participated across the study period. Of note, the UEFA club coefficient rankings based on the results of all European clubs in the UEFA club competition have not been reported due to a large proportion of teams included in the study competing in the English Championship, League One, and League Two (Table S1).

**TABLE 1:** Sample characteristics.

	Season												
	13–14	14–15	15–16	16–17	17–18	18–19	19–20	20–21	21–22	22–23	23–24	24–25	Total
Total teams (*n*)	13	18	13	19	15	18	22	15	70	71	70	72	416
Teams providing match exposure (*n*)	13	16	12	19	14	17	22	15	69	71	70	72	410
Match exposure (*h*)	10231.5	13205.2	9828.2	14789.2	11365.5	14001.2	17687.4	13117.1	62058.9	63315.4	64621.9	64306.3	358527.8
Match injuries (*n*)	267	323	202	401	276	391	440	283	1638	1617	1542	1557	8937
Teams providing training exposure (*n*)	9	14	10	19	14	18	20	13	65	63	64	59	368
Training exposure (*h*)	41008.8	61891.5	48436.5	85181.1	65378.1	97781.4	110358.4	60851.4	318325.8	298657.3	384596.6	355034.8	1927501.8
Training injuries (*n*)	330	324	200	395	284	373	373	267	1126	1240	1044	1093	7049

*Note:* The 21–22 season represented the point at which mandatory injury data sharing with the Injury and Illness Surveillance Programme (IISP) was implemented across EFL teams.

Abbreviations: *n*, frequency; *h*, hours.

### 2.3 Injury and Exposure Data Collection

Data were collected and analyzed based on international consensus guidelines for conducting epidemiological studies in professional football [24, 25]. A reportable injury was defined as one that occurred during club‐related activity (match‐play or training) which prevented a player from taking part in match‐play or training for 1 or more days following the occurrence [24, 25]. The retrospective categorization of injury severity, cause, onset, type, location, and diagnosis was based on consensus guidelines [24, 25] and previous research from the *FA IISP* [22, 23]. The time loss for injuries was calculated as the number of days that the player was unable to take part in regular match‐play or training. Injury information was recorded and classified by each team's medical support staff using the club's preferred electronic medical record system [26]. To categorize injury severity, the following thresholds were adhered to: 1–7 days absence (minor), 8–28 days (moderate), 29–89 days (severe), and ≥ 90 days (major). Injuries sustained outside of formal match‐play and training were excluded from the analyses.

Any formal match exposure (league/cup fixtures against another team) and all club‐directed training exposure that took place during each season were recorded by club medical support staff via the club's preferred method of collecting exposure minutes, for example, from global positioning systems (GPS) or bespoke exposure collection forms. All data were transferred directly to the research group via email in protected files with specified accessibility, processed, and stored within a secure, bespoke database alongside the injury data. Match and training exposure data could be provided on a daily, monthly or yearly basis, capturing all exposure minutes on a squad basis, or provided on an anonymized per‐player basis and aggregated when processed by the research group. Values were formed as follows:

Match Exposure: Total minutes played per player during a match in its entirety, including extra time.

Training Exposure: Reported on a squad basis, with modification information provided for players who were not involved in the ‘regular’ training session. Exposure for friendlies, warm‐ups, on‐pitch sessions, individual preparation and recovery was requested for the study dataset. Player leisure/non‐club related activities outside of structured sessions were not recorded.

Before the commencement of the study, all club medical support staff were provided with bespoke guidance documents produced by the FA and others [27], as well as individual guidance if requested. Information on why the study was taking place, definitions of each variable, and required inputs were provided, and individual practitioners were able to raise any questions via email.

### 2.4 Data Analysis

Injury incidence rate is reported as the number of injuries per 1000 h of exposure, and injury burden is reported as the total number of days absent per 1000 h of exposure, with time loss (days) reported as median severity (IQR). Where data on match or training exposure were not available, any corresponding injuries occurring during this period were excluded from injury incidence rate and burden analyses. The number of teams contributing both injury and exposure data in each season is shown in Figure S1. Any data presented without the use of exposure as a denominator (i.e., absolute injury frequency and days absent, proportional analysis, median days absent) were represented by the complete injury sample count and corresponding days absent.

Match and training incidence rate and burden estimates for all injuries were reported with accompanying 95% CIs based on estimates derived from a negative binomial generalized linear model (GLM) with event indicators and a log‐exposure offset (with a team‐cluster bootstrap employed to account for the hierarchical structure of the data). In each of the 10 000 bootstrap iterations, teams were resampled with replacement and the negative binomial GLM was refitted to the resampled data to derive incidence rate and burden estimates; confidence intervals were obtained from the 2.5th and 97.5th percentiles of the bootstrap distribution. The overall match versus training injury incidence rate ratio (IRR) and injury burden ratio (IBR) with associated 95% CIs were derived from the same cluster bootstrap; within each iteration, the ratio of the match to training estimates was computed, with 95% CIs obtained from the percentiles of the resulting ratio distribution. Effect estimates with 95% CIs excluding 1.00 indicated statistical significance.

Time‐trend analysis was performed employing a similar clustered, bootstrapped negative binomial approach to determine changes in match and training incidence rate and burden across the study period. A log‐linear negative binomial model was used within each bootstrap iteration to estimate yearly trends. Data from the 2013 to 2014, 2014 to 2015, 2015 to 2016 seasons were combined to form an aggregate of the first three seasons, reflecting the relatively small number of contributing teams in each and avoiding the use of any single season as the baseline for comparison. The percentage change per year was calculated using the formula: (exp [β]–1)*100, where the slope coefficient (β) represents the log of the yearly change of the outcome variable. Statistical significance was set at *p* < 0.05, with two‐sided *p*‐values computed from the bootstrap distribution of the slope coefficient as twice the smaller of the proportions of bootstrap slopes at or above and at or below zero.

For injury type, location, diagnosis, severity, cause, and onset, point estimates of incidence rate and burden were calculated as crude rates (observed counts divided by the corresponding exposure), aggregated across teams. Confidence intervals were derived from parametric negative binomial simulations of the observed counts. The IRR and IBR [95% CI] for comparisons between matches and training were derived from independently drawn simulations of the numerator and denominator counts, with confidence intervals obtained from the percentiles of the resulting ratio distributions. Ratio estimates with associated 95% CIs excluding 1.00 indicated statistical significance. All analyses were performed using the open‐access R software (Version 4.3.3, R Foundation for Statistical Computing, Vienna, Austria, www.r‐project.org), using the MASS package to conduct the negative binomial regression models and bootstrap analyses.

## 3 Results

A total of 15 986 injuries were recorded across 12 seasons, consisting of 8937 match injuries (56%) and 7049 training injuries (44%). There was a total of 498 108 days absent, 298 571 due to match injuries and 199 537 days absent due to training injuries. Over the 12‐year study period, total exposure was 2 286 030 h, consisting of 358 528 h in matches and 1 927 502 h in training (Table 1).

Where data on match or training exposure were not available, any corresponding injuries occurring during this period were excluded from injury incidence rate and burden analyses. Overall, 1% and 13% of all injuries for matches and training, respectively, were excluded from the incidence rate and burden calculations. This corresponded to 0.7% and 15% of all days absent due to match and training injuries, respectively, being excluded from the injury burden calculations. Details regarding injury characteristics based on data where match and training exposure were not available can be found in Tables S1–, S6.

### 3.1 Injury Incidence Rate and Burden

Match injury incidence rate (24.9 injuries/1000 h [95% CI: 24.0–25.8]) was higher than training (3.6 injuries/1000 h [95% CI: 3.3–3.8]) (IRR: 7.0 [95% CI: 6.6–7.4]). No change was observed in match injury incidence rate across the study period (−0.01%, *β* = −0.0001, *p* = 0.992) (Figure 1), but there was a 4% decrease in training injury incidence rate (*β* = −0.04, *p* < 0.001) (Figure 2). Match injury burden (835.5 days absent/1000 h [95% CI: 803.5–867.9]) was higher than training (104.1 days absent/1000 h [95% CI: 96.9–111.0]) (IBR: 8.0 [95% CI: 7.5–8.7]). There was a 5% increase in match injury burden across the study period (*β* = 0.05, *p* < 0.001) (Figure 1), but there was no change in training injury burden (1%, *β* = 0.01, *p* = 0.343) (Figure 2). Additionally, sensitivity analyses restricted to teams with repeated participation produced materially unchanged estimates and temporal trends (Table S7).

**FIGURE 1:**
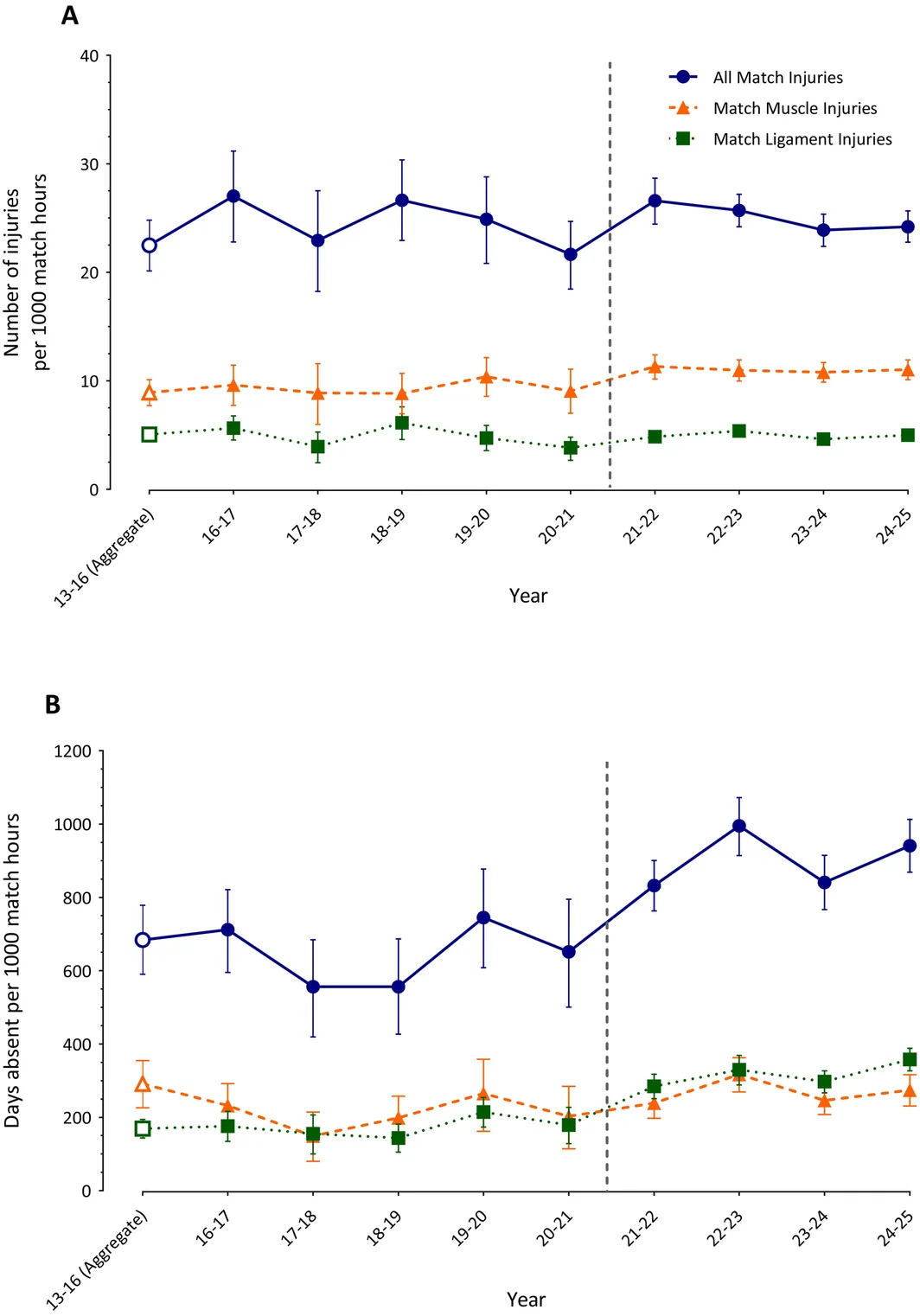
Time trend of match injury incidence rate (A) and burden (B) for all injuries, muscle injuries and ligament injuries across the 12‐year study period. The first observation represents an aggregated estimate derived from the 13 to 14, 14 to 15, 15 to 16 seasons and is indicated by an open symbol. For time‐trend analyses, this aggregated estimate was included as the initial observation in the model, followed by individual season estimates from 16 to 17 onwards. The number of contributing teams is as follows: 13–16: *N* = 41, 16–17: *N* = 19, 17–18: *N* = 14, 18–19: *N* = 17, 19–20: *N* = 22, 20–21: *N* = 15, 21–22: *N* = 69, 22–23: *N* = 71, 23–24: *N* = 70, 24–25: *N* = 72. This equates to 410 team seasons. The dotted vertical line indicates the mandatory sharing of injury data with the Injury and Illness Surveillance Programme (IISP) for EFL Teams (21–22 season). Injury incidence rate is presented as the number of injuries/1000 h, with associated 95% CIs. Injury burden is presented as the number of days absent/1000 h, with associated 95% CIs.

**FIGURE 2:**
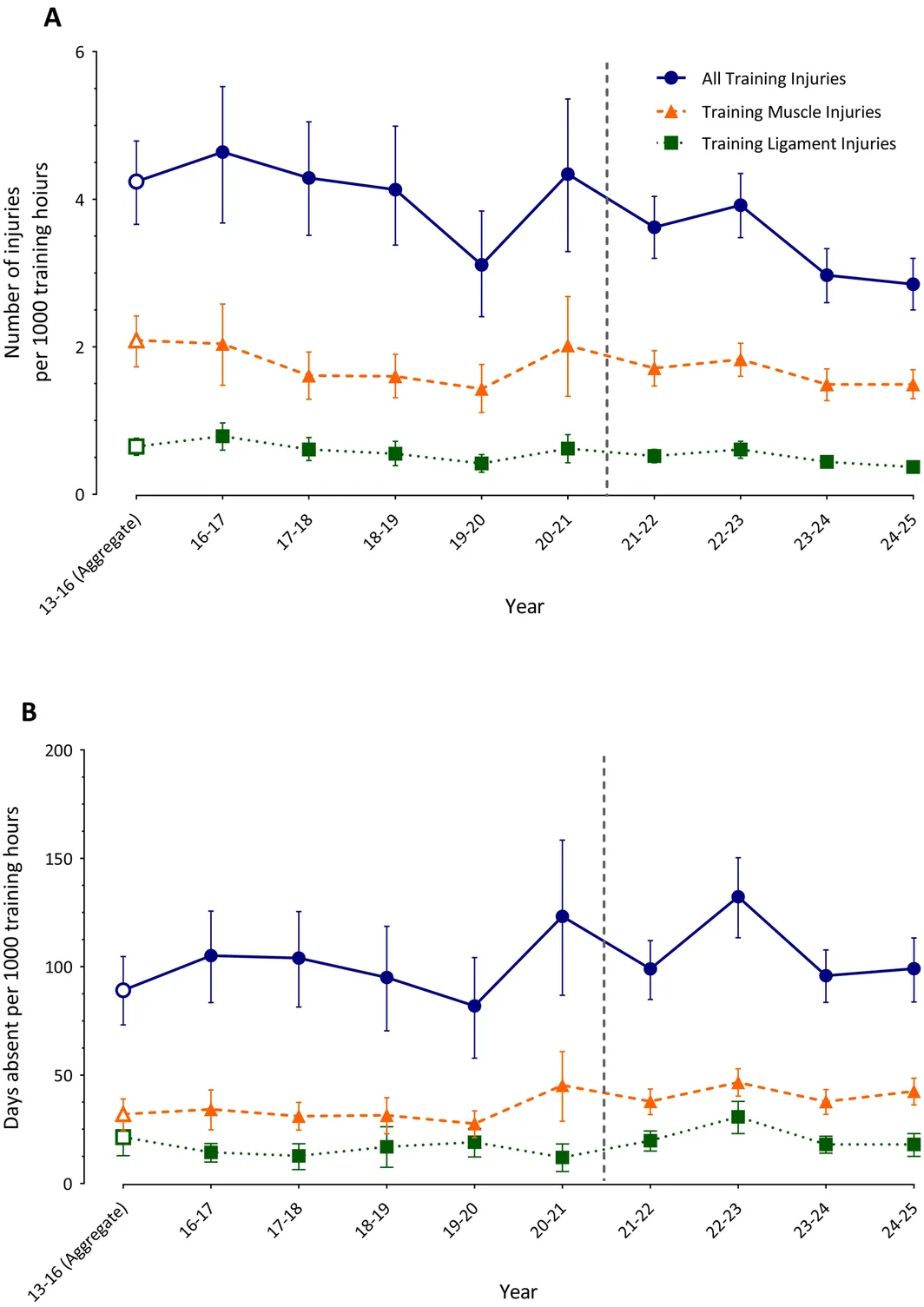
Time trend of training injury incidence rate (A) and burden (B) for all injuries, muscle injuries and ligament injuries across the 12‐year study period. The first observation represents an aggregated estimate derived from the 13 to 14, 14 to 15, 15 to 16 seasons and is indicated by an open symbol. For time‐trend analyses, this aggregated estimate was included as the initial observation in the model, followed by individual season estimates from 16 to 17 onwards. The number of contributing teams is as follows: 13–16: *N* = 33, 16–17: *N* = 19, 17–18: *N* = 14, 18–19: *N* = 18, 19–20: *N* = 20, 20–21: *N* = 13, 21–22: *N* = 65, 22–23: *N* = 63, 23–24: *N* = 64, 24–25: *N* = 59. This equates to 368 team seasons. The dotted vertical line indicates the mandatory sharing of injury data with the Injury and Illness Surveillance Programme (IISP) for EFL Teams (21–22 season). Injury incidence rate is presented as the number of injuries/1000 h, with associated 95% CIs. Injury burden is presented as the number of days absent/1000 h, with associated 95% CIs.

### 3.2 Type of Injury

Muscle and ligament injuries were the most common type of injury in both matches and training, and recorded the highest burden (Table 2). The injury incidence rate and burden reported for the types of injury were consistently higher in matches than in training. Specifically, muscle injuries were more common in matches than in training (IRR: 6.79 [3.57–13.02]), as were ligament injuries (IRR: 9.94 [5.30–18.75]). Similarly, the match injury burden of muscle (IBR: 8.08 [4.26–15.35]) and ligament injuries (IBR: 15.08 [8.09–28.7]) was greater than in training.

**TABLE 2:** Frequency, incidence rate, median severity and burden of the types of injury that occurred overall, in matches and training.

Injury type	Frequency (*n*, % of total)			Incidence rate (injuries/1000 h [95% CI])			IRR (95% CI)	Median severity (days absent [IQR])			Burden (days absent/1000 h [95% CI])			IBR (95% CI)
	All	Match	Training	All	Match	Training		All	Match	Training	All	Match	Training	
**Muscle strain/tear/rupture/cramps**	7125 (44.6%)	3779 (42.3%)	3346 (47.5%)	2.93 (1.77–4.35)	10.41 (6.46–15.44)	1.53 (0.94–2.28)	6.79 (3.57–13.02)^a^	15 (8–30)	17 (9–33)	14 (7–28)	70.90 (43.45–104.73)	271.52 (165.73–405.45)	33.58 (20.44–49.85)	8.08 (4.26–15.35)^b^
**Ligament sprain/tear/rupture**	2931 (18.3%)	1789 (20.0%)	1142 (16.2%)	1.20 (0.73–1.76)	4.95 (2.98–7.31)	0.50 (0.30–0.74)	9.94 (5.30–18.75)^a^	22 (8–54)	27 (10–59)	16 (6–43)	55.23 (33.75–81.33)	259.4 (159.47–386.62)	17.21 (10.43–25.46)	15.08 (8.09–28.70)^b^
**Tendon injury/rupture/tendinosis**	1331 (8.3%)	682 (7.6%)	649 (9.2%)	0.55 (0.33–0.82)	1.89 (1.15–2.80)	0.30 (0.18–0.44)	6.42 (3.37–12.22)^a^	25 (8–57)	29 (11–67)	19 (7–47.75)	24.95 (15.17–37.21)	97.79 (59.13–144.83)	11.40 (6.91–16.93)	8.58 (4.55–16.39)^b^
**Haematoma/contusion/bruise**	1094 (6.8%)	776 (8.7%)	318 (4.5%)	0.47 (0.28–0.69)	2.14 (1.29–3.19)	0.15 (0.09–0.23)	13.82 (7.35–26.51)^a^	5 (3–10)	5 (3–11)	5 (3–9)	4.64 (2.82–6.83)	21.55 (13.22–32.28)	1.50 (0.91–2.20)	14.40 (7.64–27.55)^b^
**Concussion**	438 (2.7%)	333 (3.7%)	105 (1.5%)	0.18 (0.11–0.28)	0.92 (0.56–1.39)	0.05 (0.03–0.07)	19.71 (10.16–39.51)^a^	8 (6–13)	8 (5–13)	7 (5–13)	2.36 (1.44–3.51)	11.75 (7.14–17.41)	0.61 (0.37–0.90)	19.34 (10.21–36.95)^b^
**Bursitis/impingement/synovitis**	419 (2.6%)	191 (2.1%)	228 (3.2%)	0.17 (0.10–0.26)	0.53 (0.31–0.81)	0.10 (0.06–0.21)	5.19 (2.66–10.28)^a^	8 (4–16)	8 (5–15.5)	8 (4–16)	2.92 (1.78–4.35)	10.50 (6.40–15.56)	1.51 (0.93–2.24)	6.95 (3.68–13.31)^b^
**Cartilage/disc/meniscus**	611 (3.8%)	258 (2.9%)	353 (5.0%)	0.24 (0.15–0.36)	0.71 (0.42–1.08)	0.15 (0.09–0.23)	4.67 (2.39–9.05)^a^	35 (12–75)	39 (15–77)	31 (11–73)	12.40 (7.55–18.40)	40.27 (24.58–59.79)	7.81 (4.68–11.53)	5.16 (2.74–9.79)^b^
**Bone fracture**	649 (4.1%)	413 (4.6%)	236 (3.3%)	0.27 (0.17–0.41)	1.14 (0.69–1.71)	0.11 (0.07–0.16)	10.35 (5.40–20.01)^a^	41.5 (19–78)	42 (18–75.75)	41 (19.75–81.5)	15.09 (9.14–22.44)	62.03 (38.08–90.98)	6.36 (3.86–9.39)	9.76 (5.14–18.62)^b^
**Pain (undiagnosed)**	360 (2.3%)	158 (1.8%)	202 (2.9%)	0.15 (0.09–0.22)	0.44 (0.26–0.66)	0.09 (0.06–0.14)	4.63 (2.38–9.18)^a^	6.5 (4–14.75)	6 (4–14)	7 (4–15)	2.27 (1.38–3.36)	6.26 (3.81–9.37)	1.53 (0.94–2.28)	4.10 (3.17–7.83)^b^
**Other**	981 (6.1%)	533 (6.0%)	448 (6.4%)	0.40 (0.24–0.59)	1.48 (0.90–2.21)	0.20 (0.12–0.30)	7.43 (3.92–14.36)^a^	15 (6–40)	15 (6–39)	15 (6–41.5)	12.40 (7.55–18.40)	43.39 (26.25–64.00)	6.64 (4.06–9.82)	6.54 (3.47–12.47)^b^
**Unspecified**	47 (0.3%)	25 (0.3%)	22 (0.3%)	0.02 (0.01–0.03)	0.07 (0.03–0.11)	0.01 (0.00–0.02)	N/A	8.5 (5–15.75)	8 (4–13.5)	10 (6–24)	0.46 (0.28–0.68)	2.35 (1.43–3.51)	0.11 (0.06–0.16)	N/A

*Note:* Frequency of injuries (*n*) and median severity (days absent, IQR) represent all injuries reported. Where corresponding exposure data were unavailable, injuries were excluded from incidence rate and burden calculations. This accounted for 1% and 13% of all injuries for matches and training, respectively, as well as 0.7% and 15% of total days absent from match and training injuries, respectively. Excluding these cases ensured accurate calculations of injury incidence rate and burden (per 1000 h).

^a^ Greater injury incidence rate in matches compared to training.

^b^ Greater injury burden in matches compared to training; determined via injury incidence rate ratios (IRR) and injury burden ratios (IBR), where effect estimates with associated 95% CIs excluding 1.00 indicate statistical significance. Injury incidence rate and burden ratios were not calculated for unspecified injuries (N/A).

The seasonal trends in muscle and ligament injury incidence rate and burden are presented in Figures 1 and 2. For muscle injuries, a 3% increase in match injury incidence rate (*β* = 0.03, *p* = 0.004) and a 3% decrease in training injury incidence rate (*β* = −0.03, *p* = 0.028) were observed. Regarding injury burden, a 10% increase was seen in matches (*β* = 0.10, *p* < 0.001) and a 4% increase was seen in training (*β* = 0.04, *p* = 0.006). For ligament injuries, time‐trend analysis showed a 5% decrease in training injury incidence rate (*β* = −0.05, *p* < 0.001). However, match injury incidence rate (−0.4%, *β* = −0.004, *p* = 0.646), match injury burden (2%, *β* = 0.02, *p* = 0.192), and training injury burden (3%, *β* = 0.03, *p* = 0.194) did not change over the course of the study period. Additionally, sensitivity analyses restricted to teams with repeated participation produced materially unchanged estimates and temporal trends (Table S7).

### 3.3 Location and Diagnosis of Injury

The thigh, knee, and ankle were the most common locations of injury in matches and training, and recorded the highest burden, with injury incidence rate and burden consistently higher in matches compared to training for all locations of injury (Tables 3 and 4).

**TABLE 3:** Frequency and incidence rate of the locations and diagnoses of injury that occurred overall, in matches and training.

Injury location	Frequency (*n*, % of total)			Incidence rate (injuries/1000 h [95% CI])			IRR (95% CI)
*Diagnosis*	All	Match	Training	All	Match	Training	
**Abdomen**	164 (1.0%)	86 (1.0%)	78 (1.1%)	0.06 (0.04–0.10)	0.23 (0.13–0.36)	0.03 (0.02–0.05)	7.06 (3.41–14.92)^a^
**Achilles tendon**	246 (1.5%)	120 (1.3%)	126 (1.8%)	0.10 (0.06–0.15)	0.33 (0.20–0.51)	0.06 (0.03–0.09)	6.03 (3.05–11.74)^a^
**Ankle**	2118 (13.2%)	1211 (13.6%)	907 (12.9%)	0.86 (0.53–1.29)	3.35 (2.04–5.00)	0.40 (0.24–0.59)	8.39 (4.40–16.06)^a^
*Lateral ankle ligament*	743 (4.6%)	422 (4.7%)	321 (4.6%)	0.31 (0.19–0.46)	1.17 (0.71–1.76)	0.15 (0.09–0.22)	7.97 (4.18–15.37)^a^
*Deltoid ankle ligament*	225 (1.4%)	136 (1.5%)	89 (1.3%)	0.09 (0.05–0.13)	0.37 (0.21–0.57)	0.04 (0.02–0.06)	10.21 (5.06–20.89)^a^
**Arm**	34 (0.2%)	18 (0.2%)	16 (0.2%)	0.01 (0.01–0.02)	0.05 (0.02–0.09)	0.01 (0.00–0.01)	6.91 (2.51–19.35)^a^
**Foot/toe**	688 (4.3%)	446 (5.0%)	242 (3.4%)	0.29 (0.18–0.43)	1.22 (0.73–1.83)	0.12 (0.07–0.17)	10.54 (5.49–20.50)^a^
**Head/face/neck/cervical spine**	634 (4.0%)	476 (5.3%)	158 (2.2%)	0.27 (0.16–0.40)	1.32 (0.80–1.97)	0.07 (0.04–0.11)	18.56 (9.65–36.76)^a^
*Concussion*	438 (2.7%)	333 (3.7%)	105 (1.5%)	0.18 (0.11–0.28)	0.92 (0.56–1.39)	0.05 (0.03–0.07)	19.71 (10.16–39.51)^a^
*Head/face/neck injury (excluding concussion)*	191 (1.2%)	141 (1.6%)	50 (0.7%)	0.08 (0.05–0.12)	0.39 (0.23–0.60)	0.02 (0.01–0.04)	17.23 (8.50–36.84)^a^
**Hip/groin**	980 (6.1%)	450 (5.0%)	530 (7.5%)	0.40 (0.24–0.60)	1.24 (0.75–1.84)	0.24 (0.15–0.36)	5.10 (2.68–9.72)^a^
*Hip/groin muscle/tendon*	612 (3.8%)	272 (3.0%)	340 (4.8%)	0.25 (0.15–0.37)	0.74 (0.45–1.12)	0.16 (0.09–0.23)	4.78 (2.49–9.38)^a^
**Knee**	2181 (13.6%)	1204 (13.5%)	977 (13.9%)	0.88 (0.54–1.31)	3.33 (2.04–4.96)	0.43 (0.26–0.64)	7.83 (4.15–15.06)^a^
*Anterior cruciate (ACL)*	180 (1.1%)	142 (1.6%)	38 (0.5%)	0.08 (0.05–0.12)	0.40 (0.23–0.60)	0.02 (0.01–0.03)	23.86 (11.47–52.74)^a^
*Medial collateral (MCL)*	507 (3.2%)	305 (3.4%)	202 (2.9%)	0.20 (0.12–0.30)	0.83 (0.50–1.24)	0.08 (0.05–0.13)	9.86 (5.13–19.27)^a^
*Meniscal/cartilage*	425 (2.7%)	190 (2.1%)	235 (3.3%)	0.17 (0.10–0.25)	0.53 (0.31–0.79)	0.10 (0.06–0.15)	5.26 (2.72–10.28)^a^
**Low back**	474 (3.0%)	173 (1.9%)	301 (4.3%)	0.19 (0.11–0.28)	0.47 (0.28–0.71)	0.13 (0.08–0.20)	3.56 (1.83–7.04)^a^
**Lower leg**	1759 (11.0%)	944 (10.6%)	815 (11.6%)	0.73 (0.44–1.08)	2.62 (1.58–3.90)	0.37 (0.23–0.56)	7.00 (3.67–13.51)^a^
*Triceps surae*	1393 (8.7%)	713 (8.0%)	680 (9.6%)	0.57 (0.35–0.85)	1.97 (1.19–2.94)	0.31 (0.19–0.46)	6.32 (3.34–12.21)^a^
**Pelvis/sacrum**	276 (1.7%)	128 (1.4%)	148 (2.1%)	0.11 (0.07–0.17)	0.35 (0.21–0.54)	0.07 (0.04–0.10)	5.17 (2.63–10.24)^a^
**Shoulder/clavicle**	326 (2.0%)	230 (2.6%)	96 (1.4%)	0.14 (0.08–0.21)	0.64 (0.38–0.95)	0.04 (0.02–0.07)	14.42 (7.34–28.74)^a^
**Sternum/ribs/upper back**	106 (0.7%)	68 (0.8%)	38 (0.5%)	0.05 (0.03–0.07)	0.19 (0.11–0.29)	0.02 (0.01–0.03)	9.88 (4.69–21.84)^a^
**Thigh**	5749 (36.0%)	3269 (36.6%)	2480 (35.2%)	2.38 (1.44–3.51)	9.02 (5.50–13.52)	1.14 (0.69–1.71)	7.90 (4.15–15.02)^a^
*Hamstring muscle/tendon*	3192 (20.0%)	2023 (22.6%)	1169 (16.6%)	1.33 (0.82–1.96)	5.58 (3.40–8.36)	0.53 (0.32–0.80)	10.44 (5.55–19.74)^a^
*Quadriceps muscle/tendon*	1150 (7.2%)	457 (5.1%)	693 (9.8%)	0.47 (0.29–0.70)	1.27 (0.76–1.89)	0.32 (0.19–0.47)	3.97 (2.08–7.60)^a^
*Adductor muscle/tendon*	1061 (6.6%)	536 (6.0%)	525 (7.4%)	0.44 (0.27–0.66)	1.48 (0.88–2.20)	0.25 (0.15–0.37)	5.99 (3.12–11.32)^a^
**Wrist/hand/finger/thumb**	204 (1.3%)	91 (1.0%)	113 (1.6%)	0.09 (0.05–0.13)	0.25 (0.15–0.39)	0.05 (0.03–0.08)	4.70 (2.30–9.59)^a^
**Unspecified**	47 (0.3%)	23 (0.3%)	24 (0.3%)	0.02 (0.01–0.03)	0.06 (0.03–0.11)	0.01 (0.01–0.02)	N/A

*Note:* Frequency of injuries (*n*) represents all injuries reported. Where corresponding exposure data were unavailable, injuries were excluded from incidence rate calculations. This accounted for 1% and 13% of all injuries for matches and training, respectively. Excluding these cases ensured accurate calculations of injury incidence rate (per 1000 h). Lower limb muscle/tendon categories exclude haematoma/contusion/bruise injuries.

^a^ Greater injury incidence rate in matches compared to training; determined via injury incidence rate ratios (IRR), where effect estimates with associated 95% CIs excluding 1.00 indicate statistical significance. Injury incidence rate ratio was not calculated for unspecified injuries (N/A).

**TABLE 4:** Median severity and injury burden of the locations and diagnoses of injury that occurred overall, in matches and training.

Injury location	Frequency (*n*, % of Total)			Median severity (days absent [IQR])			Burden (days absent/1000 h [95% CI])			IBR (95% CI)
*Diagnosis*	All	Match	Training	All	Match	Training	All	Match	Training	
**Abdomen**	164 (1.0%)	86 (1.0%)	78 (1.1%)	13 (6.5–30)	11.5 (5–22)	15 (8–34)	1.63 (1.00–2.44)	5.20 (3.14–7.75)	0.97 (0.58–1.44)	5.38 (2.85–10.30)^a^
**Achilles tendon**	246 (1.5%)	120 (1.3%)	126 (1.8%)	26 (9–72)	28 (10–95)	24 (7–55)	6.21 (3.76–9.14)	25.32 (15.38–37.90)	2.65 (1.61–3.94)	9.54 (5.14–18.06)^a^
**Ankle**	2118 (13.2%)	1211 (13.6%)	907 (12.9%)	16 (6–40)	19 (7–44)	13 (6–34)	25.96 (15.86–38.37)	106.19 (65.73–157.22)	11.04 (6.74–16.36)	9.62 (5.17–18.01)^a^
*Lateral ankle ligament*	743 (4.6%)	422 (4.7%)	321 (4.6%)	15 (7–33)	16 (8–33)	14 (6–31)	7.68 (4.67–11.34)	29.28 (17.80–43.38)	3.66 (2.22–5.44)	7.97 (4.18–15.37)^a^
*Deltoid ankle ligament*	225 (1.4%)	136 (1.5%)	89 (1.3%)	17 (7–39.5)	20 (8–47.5)	13 (7–27)	2.86 (1.74–4.23)	13.83 (8.52–20.51)	0.82 (0.50–1.23)	16.83 (8.91–31.97)^a^
**Arm**	34 (0.2%)	18 (0.2%)	16 (0.2%)	28 (5–70.25)	28 (5–70.25)	28 (7–67.25)	0.62 (0.38–0.92)	2.18 (1.32–3.25)	0.33 (0.20–0.49)	6.65 (3.50–12.81)^a^
**Foot/toe**	688 (4.3%)	446 (5.0%)	242 (3.4%)	14 (5–43)	15 (5–43)	12.5 (5–39.5)	8.9 (5.40–13.29)	38.31 (23.42–56.51)	3.43 (2.10–5.11)	11.18 (5.93–21.38)^a^
**Head/face/neck/cervical spine**	634 (4.0%)	476 (5.3%)	158 (2.2%)	8 (5–13)	8 (5–13)	7 (5–13)	3.83 (2.34–5.69)	18.18 (11.05–26.96)	1.16 (0.72–1.72)	15.68 (8.29–29.96)^a^
*Concussion*	438 (2.7%)	333 (3.7%)	105 (1.5%)	8 (6–13)	8 (6–13)	7 (5–13)	2.36 (1.44–3.51)	11.75 (7.14–17.41)	0.61 (0.37–0.90)	19.34 (10.21–36.95)^a^
*Head/face/neck injury (excluding concussion)*	191 (1.2%)	141 (1.6%)	50 (0.7%)	7 (3–16.5)	6 (3–15)	7 (4–21.5)	1.37 (0.82–2.01)	6.28 (3.78–9.32)	0.45 (0.27–0.67)	13.89 (7.40–26.18)^a^
**Hip/groin**	980 (6.1%)	450 (5.0%)	530 (7.5%)	10 (5–23)	11 (5–22)	10 (5–24.75)	8.51 (5.23–12.72)	24.78 (15.03–36.63)	5.48 (3.32–8.19)	4.52 (2.43–8.68)^a^
*Hip/groin muscle/tendon*	612 (3.8%)	272 (3.0%)	340 (4.8%)	10 (5–21)	11 (6–20)	10 (5–22)	4.70 (2.48–6.98)	13.03 (7.96–19.43)	3.15 (1.93–4.69)	4.13 (2.20–7.87)^a^
**Knee**	2181 (13.6%)	1204 (13.5%)	977 (13.9%)	26 (8–64)	33 (9–74)	18 (6–52)	50.23 (30.68–74.83)	224.63 (136.46–330.87)	17.79 (10.84–26.34)	12.63 (6.65–23.96)^a^
*Anterior cruciate (ACL)*	180 (1.1%)	142 (1.6%)	38 (0.5%)	275 (201.75–304)	277 (203.5–309)	274 (176–292)	18.16 (11.17–27.17)	97.80 (59.64–143.76)	3.45 (2.09–5.09)	28.33 (15.12–53.22)^a^
*Medial collateral (MCL)*	507 (3.2%)	305 (3.4%)	202 (2.9%)	28 (10–55)	36 (15–59)	18 (7–44)	7.82 (4.77–11.56)	36.15 (21.99–53.49)	2.55 (1.56–3.77)	14.15 (7.52–26.67)^a^
*Meniscal/cartilage*	425 (2.7%)	190 (2.1%)	235 (3.3%)	41 (16–83.5)	44.5 (20–85.75)	36 (13.5–81)	9.98 (6.05–14.71)	33.09 (20.46–48.92)	5.68 (3.44–8.46)	5.83 (3.09–11.13)^a^
**Low back**	474 (3.0%)	173 (1.9%)	301 (4.3%)	9 (5–26)	9 (5–26)	9 (4–26)	4.18 (2.53–6.21)	10.16 (6.19–14.99)	3.07 (1.88–4.48)	3.31 (1.74–6.32)^a^
**Lower leg**	1759 (11.0%)	944 (10.6%)	815 (11.6%)	18 (8–36)	18 (9–37)	18 (7–34)	19.41 (11.90–28.72)	73.59 (45.06–109.63)	9.33 (5.67–13.92)	7.88 (4.21–14.87)^a^
*Triceps surae*	1393 (8.7%)	713 (8.0%)	680 (9.6%)	19 (10–35)	19 (11–36)	19 (9–33)	14.43 (8.92–21.42)	51.13 (31.47–75.58)	7.61 (4.63–11.28)	6.72 (3.59–12.63)^a^
**Pelvis/sacrum**	276 (1.7%)	128 (1.4%)	148 (2.1%)	8 (4–15)	7 (4–12)	8 (4.5–18)	1.71 (1.04–2.55)	4.34 (2.68–6.44)	1.22 (0.74–1.81)	3.54 (1.88–6.71)^a^
**Shoulder/clavicle**	326 (2.0%)	230 (2.6%)	96 (1.4%)	21 (8–47)	20 (8–52)	27.5 (8.25–45.75)	4.90 (2.99–7.30)	21.53 (13.11–31.96)	1.81 (1.08–2.67)	11.88 (6.34–22.32)^a^
**Sternum/ribs/upper back**	106 (0.7%)	68 (0.8%)	38 (0.5%)	10 (4–21)	12 (5.75–25)	5 (3.25–10.75)	0.73 (0.45–1.09)	3.50 (2.13–5.23)	0.21 (0.13–0.32)	16.54 (8.68–31.30)^a^
**Thigh**	5749 (36.0%)	3269 (36.6%)	2480 (35.2%)	16 (8–33)	18 (9–37)	14 (7–29)	63.99 (38.67–94.83)	259.59 (159.74–383.81)	27.61 (16.87–41.12)	9.40 (5.02–17.91)^a^
*Hamstring muscle/tendon*	3192 (20.0%)	2023 (22.6%)	1169 (16.6%)	19 (10–39.75)	21 (11–43)	17 (8–34)	40.37 (24.80–59.72)	180.81 (110.58–269.53)	14.24 (8.74–21.06)	12.70 (6.70–24.17)^a^
*Quadriceps muscle/tendon*	1150 (7.2%)	457 (5.1%)	693 (9.8%)	17 (9–36)	19 (10–40)	16 (8–32.5)	13.45 (8.14–19.92)	39.87 (24.60–59.60)	8.54 (5.22–12.79)	4.67 (2.48–8.91)^a^
*Adductor muscle/tendon*	1061 (6.6%)	536 (6.0%)	525 (7.4%)	12 (6–21)	14 (8–23)	10 (6–19)	8.48 (5.15–12.55)	31.08 (18.96–45.93)	4.27 (2.64–6.32)	7.27 (3.87–13.93)^a^
**Wrist/hand/finger/thumb**	204 (1.3%)	91 (1.0%)	113 (1.6%)	18 (6–43.5)	13 (4–42)	19 (7.75–46.5)	2.62 (1.61–3.88)	6.76 (4.13–10.07)	1.85 (1.14–2.73)	3.66 (1.93–6.94)^a^
**Unspecified**	47 (0.3%)	23 (0.3%)	24 (0.3%)	11 (4.75–31.25)	11 (4–44)	11 (5.5–27)	0.69 (0.42–1.02)	2.80 (1.69–4.15)	0.30 (0.18–0.44)	N/A

*Note:* Frequency of injuries (*n*) and median severity [days absent, IQR] represent all injuries reported. Where corresponding exposure data were unavailable, injuries were excluded from incidence rate and burden calculations. This accounted for 1% and 13% of all injuries for matches and training, respectively, as well as 0.7% and 15% of total days absent from match and training injuries, respectively. Excluding these cases ensured accurate calculations of injury incidence rate and burden (per 1000 h). Lower limb muscle/tendon categories exclude haematoma/contusion/bruise injuries.

^a^ Greater injury burden in matches compared to training, determined by injury burden ratios (IBR), where effect estimates with associated 95% CIs excluding 1.00 indicate statistical significance. Injury burden ratio was not calculated for unspecified injuries (N/A).

Hamstring injury was the most common diagnosis of injury in both matches (5.58/1000 h [3.40–8.36]) and training (0.53/1000 h [0.32–0.80]), followed by triceps surae injury in matches (1.97/1000 h [1.19–2.94]) and quadriceps injury in training (0.32/1000 h [0.19–0.47]). Hamstring injury also recorded the highest injury burden of any injury diagnoses in both matches (180.81 days absent/1000 h [110.58–269.53]) and training (14.24 days absent/1000 h [8.74–21.06]), followed by ACL injury in matches (97.80 days absent/1000 h [59.64–143.76]) and quadriceps injury in training (8.54 days absent/1000 h [5.22–12.79]) (Table 3).

The incidence rate and burden of all injury diagnoses investigated in the present study were higher in matches than in training. The largest differences in both injury incidence rate and burden between matches and training were observed for ACL injury (IRR: 23.86 [11.47–52.74]; IBR: 28.33 [15.12–53.22]) and concussion injury (IRR: 19.71 [10.16–39.51]; IBR: 19.34 [10.21–36.95]). Of the major muscle groups investigated in the present study, hamstring injuries exhibited the largest difference in injury incidence rate (IRR: 10.44 [5.55–19.74]) and burden (IBR: 12.70 [6.70–24.17]) between matches and training (Table 4).

### 3.4 Severity, Cause, and Onset of Injury

Overall, moderate severity injuries were the most common (8–28 days, 2.66 injuries/1000 h [1.62–3.95], 40%), with most injuries occurring via non‐contact mechanisms (3.87 injuries/1000 h [2.38–5.76], 66%) and acute onset (4.38 injuries/1000 h [2.68–6.58], 80%). The injury incidence rates for all severity categories, causes of injury (contact/non‐contact) and onset of injury (acute/gradual) were all higher in matches than training. The largest match‐to‐training differences were observed for major injuries (IRR: 10.05 [5.28–19.32]), contact injuries (IRR: 14.76 [7.76–28.29]), and acute injuries (IRR: 9.62 [5.10–18.38]) (Table 5).

**TABLE 5:** Frequency and incidence rate of the severity, cause and onset of injury overall, in matches and training.

	All		Match		Training		IRR (95% CI)
	No. of Injuries (*n*, %)	Incidence Rate (Injuries/1000 *h* [95% CI])	Match No. of Injuries (*n*, %)	Match Incidence Rate [Injuries/1000 h (95% CI)]	Training No. of Injuries (*n*, %)	Training Incidence Rate [Injuries/1000 h (95% CI)]	
**Severity**	**(*n* = 15 919)**		**(*n* = 8899)**		**(*n* = 7020)**		
Minor	4417 (28%)	1.81 (1.10–2.68)	2226 (25%)	6.14 (3.68–9.10)	2191 (31%)	1.00 (0.61–1.50)	6.13 (3.24–11.71)^a^
Moderate	6430 (40%)	2.66 (1.62–3.95)	3635 (41%)	10.05 (6.19–14.91)	2795 (40%)	1.28 (0.78–1.90)	7.82 (4.17–14.81)^a^
Severe	3966 (25%)	1.63 (0.98–2.39)	2364 (27%)	6.54 (3.99–9.60)	1602 (23%)	0.71 (0.44–1.07)	9.17 (4.89–17.25)^a^
Major	1106 (7%)	0.45 (0.28–0.67)	674 (8%)	1.87 (1.12–2.79)	432 (6%)	0.19 (0.11–0.28)	10.05 (5.28–19.32)^a^
**Cause**	**(*n* = 14 247)**		**(*n* = 8252)**		**(*n* = 5995)**		
Contact	4669 (33%)	1.96 (1.20–2.90)	3325 (40%)	9.18 (5.65–13.65)	1344 (22%)	0.62 (0.38–0.94)	14.76 (7.76–28.29)^a^
Non‐Contact	9345 (66%)	3.87 (2.38–5.76)	4840 (59%)	13.39 (8.13–19.86)	4505 (75%)	2.10 (1.28–3.15)	6.36 (3.35–12.06)^a^
Cumulative	233 (2%)	0.09 (0.05–0.13)	87 (1%)	0.23 (0.13–0.36)	146 (2%)	0.06 (0.04–0.09)	3.72 (1.85–7.43)^a^
**Onset**	**(*n* = 13 221)**		**(*n* = 7723)**		**(*n* = 5498)**		
Acute	10 566 (80%)	4.38 (2.68–6.58)	6484 (84%)	17.93 (11.08–26.35)	4082 (74%)	1.86 (1.14–2.77)	9.62 (5.10–18.38)^a^
Gradual	2655 (20%)	1.07 (0.66–1.60)	1239 (16%)	3.41 (2.09–5.05)	1416 (26%)	0.63 (0.38–0.94)	5.39 (2.82–10.18)^a^

*Note:* Frequency of injuries (n) represents all injuries with a known severity, onset and cause of injury. Therefore, where data were unspecified, the injuries were removed from the total number of injuries. Additionally, where corresponding exposure data were unavailable, injuries were excluded from incidence rate calculations. This accounted for 1% and 13% of all injuries for matches and training, respectively. Excluding these cases ensured accurate calculations of injury incidence rate (per 1000 h).

^a^ Greater injury incidence rate in matches compared to training; determined by injury incidence rate ratios (IRR), where effect estimates with associated 95% CIs excluding 1.00 indicate statistical significance.

## 4 Discussion

The current study provides an up‐to‐date, comprehensive examination of injury epidemiology in English men's professional domestic football. The study aggregated data from clubs playing in the English Premier League, Championship, League One, and League Two across 12 seasons (2013–2014 to 2024–2025). Findings suggest the incidence rate and burden of match injuries were greater than that of training across all outcome measures. Cohort‐level temporal trends across the study period suggested there was a 4% reduction in training injury incidence rate and a 5% increase in match injury burden per season. Muscle and ligament injuries were the most common types of injury and recorded the highest injury burden. Cohort‐level temporal trends suggested there was an increase in match incidence rate, match burden, and training burden of muscle injuries across the study period. Finally, the injury diagnosis that was most common and recorded the highest burden in English men's professional domestic football was hamstring injury.

### 4.1 Match Injury Incidence Rate and Burden Are Greater Than Training

Match injury incidence rate was seven times higher than training in the present study (24.9 vs. 3.6 injuries/1000 h), which aligns with previous reports across professional football (36.0 vs. 3.7 injuries/1000 h) [28], specifically in large‐scale European research [18] and in MLS [19] therefore demonstrating a consistently greater incidence rate of injuries in matches. This heightened incidence rate is also aligned with a markedly greater injury burden compared to training (835.5 vs. 104.1 days absent/1000 h), again coinciding with previous research in professional football (504.6 vs. 60.5 days absent/1000 h) [18]. Not only this, but the present study identified large discrepancies between match‐specific estimates of type, location, onset, and cause of injury compared with training, further highlighting the greater incidence rate and burden of injury in matches. Specifically, concussion and ACL injuries were observed to have large differences in injury incidence rate and burden between matches and training, aligning with previous research [29, 30], with hamstring injuries representing the muscle group with the greatest differences, particularly regarding injury severity, which supports previous research in MLS players [21]. The high physical demands of professional football match‐play have been established, such as high‐intensity efforts, increased likelihood of contact situations, complex technical movements, and the unpredictable nature of match‐specific actions. It is feasible to suggest that training may not replicate the demands of match‐play, as shown by, for example, the significantly lower incidence rate of contact injuries in training (IRR: 14.76; Table 5). Additionally, the inherent situational unpredictability of match‐play environments compared with the more controlled nature of training environments may further contribute to these differences. Collectively, these factors may underpin the disparities observed between match and training estimates in the present study. Specifically, this may result in less time to implement football‐specific drills, exercise modalities, and injury prevention work at thresholds which can have a protective effect against the higher match demands and injury risk. The methods, usability, and informative outcome measures of load monitoring have been widely assessed [31], with the benefits and barriers perceived by coaches and practitioners evident [32]. However, the differences between match‐play and training demands, and their relationship with injury risk and burden in men's professional football, are inconclusive. Training constitutes a large proportion of players' footballing exposure, representing many components such as on‐pitch sessions, conditioning, rehabilitation, and recovery. Therefore, critically evaluating the usefulness of current monitoring tools and preparation programmes in relation to each training component may better prepare players for the demands of match‐play, and remains a consideration for future injury prevention strategies.

### 4.2 Seasonal Trends in Matches

Interestingly, match injury incidence rate did not change across the 12‐year study period, which contrasts the 3% decrease reported over 18 years in the men's professional European cohort (UEFA ECIS) [18], and the descriptively reported decline over the course of 6 seasons in the MLS [19]. The lack of change in English professional football is apparent, despite alterations that could positively impact injury incidence rate in matches, such as rule and policy updates, technological advancements, improved diagnostic abilities, and injury prevention knowledge [18, 33]. Conversely, factors that could negatively impact injury incidence rate, such as fixture density [7, 8, 9], and high‐intensity physical demands specific to English football [6], have also not appeared to have influenced match injury incidence rate. However, a 5% increase in match injury burden per season was reported. Although the incidence rate of injuries remains stable, the shift in match‐play characteristics toward higher intensity actions and overall greater demands means it is reasonable to suggest that such changes have increased the burden of injuries over time.

Importantly, these alterations in match‐play characteristics may also have a direct role in the patterns and temporal trends observed for match‐related muscle injuries. The predominance of muscle injuries in the present study supports previous epidemiological data [28], specifically in smaller samples in English professional football [1, 16, 17]. In comparison to the 18‐year UEFA ECIS [18], muscle injury incidence rate in matches is similar to the present study, although muscle injury burden is greater in English men's domestic football than European domestic football (271.52 vs. 178.40 days absent/1000 h). Despite overall match injury incidence rate remaining stable, a 3% increase in match muscle injury incidence rate per season was observed, accompanied by a 10% increase in muscle injury burden. This is in contrast with the findings from the 18‐year UEFA ECIS, where no significant trends were identified [18]. It is important to note that the 18‐year UEFA ECIS reflects injury patterns between 2000 and 2019, including a limited number of English‐based teams. The present study extends surveillance data through to 2025 and provides a more comprehensive and representative overview of injury patterns within English professional football, particularly in the context of seasons post‐pandemic. This difference in study periods and population may explain the contrasting findings between the UEFA ECIS and the present study. Supporting this notion, more recent research from 2001 to 2022 in European football has identified rising trends in specific muscle injuries, particularly those affecting the hamstrings [20]. The observations may also partly be influenced by the intensified external demands imposed in English professional football over the last decade, given the increasing volumes of repeated sprinting, high‐speed running, accelerations and decelerations [6, 34], which can lead to injury. For example, previous research has referenced an increased risk of muscle injury during match‐play due to incremental sprint distances covered over one minute [35]. While the mechanisms are not completely established, the increasing temporal trends could be related to greater high‐intensity movement patterns, placing significant strain on muscle tissues.

As a minimum, English Football League teams typically played 46 games per season across an approximate 40‐week competitive season (additional fixtures from 3 cup competitions). Fixtures are scheduled for 1–2 times per week, occasionally playing 2–3 games in congested periods, with no enforced winter break. Fixture density has been associated with match muscle injury incidence rate, where increases have been shown in periods of excessive match load sequences [7], with a 20% lower injury incidence rate after 6 days of recovery compared to 3 days [8]. This could be attributed to player fatigue and resultant alterations in physiological [36] and biomechanical [37] muscle properties, influencing injury incidence rate and its associated burden. To date, previous research examining the demands, running characteristics, and fixture density has been conducted in English Premier League teams. However, the quantification of these variables was not reported in the present study and their association with injury in the English Championship to League Two has not been recently established [34], providing an interesting avenue for future research investigating how fixture density is associated with injury.

### 4.3 Seasonal Trends in Training

Training injury incidence rate was 3.6 injuries/1000 h, demonstrating a 4% decrease per season across the study period, while training injury burden remained stable. Similar findings were also reported in both the 18‐year UEFA ECIS and across 6 seasons in the MLS [18, 19], both reporting an incidence rate of 3.4 injuries/1000 h and a declining trend over the course of the investigations. The knowledge of exercise‐based prevention strategies has increased, and their effectiveness in reducing injuries has been reported [38]. Enhanced monitoring technologies, diagnostic capabilities, and better collaboration between coaches and medical support staff are other external factors that may have played a role in the reduction of training injuries [18, 39]. Given that training is more controllable and amenable to injury prevention [28], and injury incidence rate is much lower than in match‐play, this may suggest a more cautious approach being adopted across the study period. This includes introducing more training modifications, prolonged rehabilitation times and an emphasis on warm‐up and recovery adherence. However, quantifying the influence of player management and monitoring on training injury incidence rate and squad availability in English professional football requires further examination to confirm these explanations.

The 4% increase in training muscle injury burden, despite conversely reporting a 3% decrease in incidence rate, is an interesting finding. Training muscle injury burden is determined by injury incidence rate, injury severity, and total exposure. Due to training muscle injury incidence rate decreasing, it could be suggested that muscle‐related training injuries are becoming more severe. This could be a result of higher‐grade injuries extending time to return to play [40], more specifically, the repeated identification of intramuscular tendon involvement. This disruption in the muscle has been associated with longer time to return to play following, for example, hamstring injuries [41], due to more rigorous rehabilitation processes and cautious approaches employed by medical practitioners to avoid re‐injury. It is also not uncommon for injuries of this nature to require surgical interventions [42]. While speculative, these physiological factors may have led to an increase in training muscle injury burden across the study period.

Additionally, muscle injury incidence rate in training was similar to the 18‐year UEFA ECIS [18], yet injury burden in training is greater in English men's domestic football (33.58 vs. 20.0 days absent/1000 h). The reason for this remains unclear; however, it could be due to the higher intensity demands of modern match‐play [6, 34], and multiple internal (e.g., range of motion, physiological features) and external factors (e.g., workload, fixture schedule and surface) [43].

With reference to external factors, a combination of fixture density and match‐related fatigue may play a role in the increasing muscle injury burden in training. Neuromuscular fatigue could be elevated across a competitive week, with it being shown that symptoms, performance detriments, and objective soreness can be present for up to 72 h post‐match [36]. If players are undertaking training with high residual fatigue and limited preparation time is available to clubs, muscle tissue may be more predisposed to a severe injury. However, the durations of injury absence during short periods of fixture density have shown no differences [9]. Clearly, there is uncertainty around the overarching effects, and there are real‐world barriers to monitoring, workload recommendations and data collection, including time, technological and human resources, and medical training or expertise [44]. This can specifically impact the monitoring of post‐match fatigue. Subsequently, this may encourage a shift toward more precautionary return‐to‐play approaches, without changing diagnostic incidence rates.

### 4.4 Location and Diagnosis

The thigh, knee, and ankle were the most common locations of injury in matches and training and recorded the highest burden, which is a consistent finding of previous epidemiological research examining football injuries [28], albeit with some variation in which is the single most common between studies. When further interpreting the present data, the patterns and trends observed for muscle injuries across the study period, particularly located at the thigh, are underpinned by the scope of injury diagnoses. This is confirmed by the commonality and high burden of hamstring injuries, specifically, but also triceps surae and quadriceps injuries noted in matches and training (Tables **3** and **4**). Therefore, this suggests that these diagnoses require the most attention from medical practitioners, highlighting the need for specified epidemiological research to reduce their inevitable negative impact on availability, team performance, and financial burden [2, 3, 4].

Specifically, hamstring injuries are of growing concern in men's professional football. This is supported by evidence showing an increase in both injury incidence rate and overall burden between 2014 and 2022 in European cohorts [20], alongside a rise in the average time to return to play in Major League Soccer from 13.6 days between 2010 and 2015, increasing to 20.6 days between 2016 and 2021 [21]. However, although MLS data suggest increased recovery time, the reported injury rate remained relatively stable (16.93% vs. 15.67%). Notably, this rate was calculated as the proportion of players sustaining a hamstring injury relative to the total number of MLS players, which limits direct comparability with UEFA ECIS data and the findings of the present study.

The present study also confirms the findings of previous research in smaller samples in English professional football [16]. Meanwhile, hamstring injury incidence rate is similar to previous reports in European football [20], whereas injury burden is considerably greater in English men's professional football in both matches and training. Despite diagnostic abilities and knowledge of hamstring injury etiology continuously increasing, with research encouraging the implementation of prevention strategies (i.e., Nordic Hamstring Exercise) [45], it remains the most common injury in men's professional football, accumulating the highest burden. Further research is required to investigate hamstring injury trends, injury‐site and grade‐specific patterns, activities at the time of injury (i.e., accelerations, decelerations), and the adoption of and compliance with injury prevention strategies. This will encourage interdisciplinary discussions around their patterns and reliably inform future applied practice.

## 5 Limitations

The present study provides the most comprehensive examination of injury epidemiology in English men's professional domestic football; however, it is not without limitations. Firstly, the present study is a retrospective analysis of prospectively collected surveillance data, which identifies significant trends and patterns in the current cohort. However, attributing any findings to causative factors is beyond the scope of the present study, where only informed explanations have been provided.

Secondly, the number of teams contributing data varied between seasons (Table 1; Figure S1; Table S2). This is partly because the FA IISP did not become mandatory until the 2021–2022 season. Evidently, there was a smaller number of teams recruited between 2013 and 2021, including an average of 3, 16, 12, and 11 teams per season in the Premier League, Championship, League One, and League Two, respectively. This may have influenced the incidence rate and burden estimates for the seasons during this period, and therefore the observed seasonal trends. Nevertheless, eight seasons of epidemiological data with four consecutive seasons of mandated data from the 2021 to 2022 season onwards, representing an average of 17 teams per season, make the present study the most comprehensive analysis of injuries in English men's professional football to date. Additionally, only 7% of unique teams contributed data for 1 season, and sensitivity analyses restricted to teams participating for ≥ 2, ≥ 3, and ≥ 4 seasons produced materially unchanged estimates and temporal trends (Table S7). This suggests that variation in team participation is unlikely to explain the present findings; however, it remains an important methodological consideration for future research.

Assessing inter‐ and intra‐club variability, together with player‐level changes over time, may help explain some of the temporal changes observed in the present study. However, professional football is characterized by substantial turnover of players, coaches, managers, and medical staff, meaning that club‐level continuity does not necessarily reflect continuity of the underlying players and staff. Furthermore, players were not identifiable in the study due to information being received in an anonymized format to comply with ethical and data sharing agreements. Consequently, this prevented the tracking of individual players across clubs or seasons and limited the ability to distinguish within‐player or within‐club temporal changes from broader cohort‐level trends. The absence of player‐level data also meant that characteristics such as age distribution, playing position, and injury history could not be examined. Therefore, the temporal trends reported in this study should be interpreted at the cohort level rather than the individual player level. Analyses were not stratified by league, and league‐specific temporal trends were beyond the scope of the present investigation, which was designed to provide an overview of injury epidemiology across English men's professional football. Future research should explore league‐specific trends and, if possible, investigate the relative contributions of player, squad, and organizational factors to observed changes over time.

Moreover, where corresponding exposure data were unavailable, injuries were excluded from incidence rate and burden calculations. This accounted for 1% and 13% of all injuries relating to matches and training, respectively, corresponding to 0.7% and 15% of total days absent (Table 1; Tables S2–, S6). This may have introduced some bias if the excluded injuries differed systematically from those included in the analyses. However, given the relatively small number of excluded cases, any impact is likely to have been limited. Findings from complete datasets were presented for all descriptive injury characteristics. Although inherent limitations exist when conducting research in professional football, the large sample size and status as the largest single‐country study of its kind strengthen the robustness of the findings. To further improve compliance, multiple staff contacts have been established at each individual club to optimize communication. The authors aim to continuously improve guidance and support processes to enhance adherence to data collection and improve data availability.

The data were collected and reported by medical practitioners at each club, which introduces the possibility of misclassification due to inter‐practitioner differences in injury diagnosis and reporting. Nevertheless, the IISP provides specific guidance and support to reduce inconsistencies in reporting across clubs. Additionally, given the variable level of diagnostic detail provided by medical practitioners within the present study, more granular analysis of injury characteristics, such as specific tissue involvement, was not possible. As discussed, it is well established that injuries affecting different muscle groups and of varying grades, particularly those involving intramuscular tendon disruption, are associated with prolonged time to return to play. It is feasible to suggest that these physiological factors may have influenced our findings that show an increase in training muscle injury burden while incidence rate has decreased. However, this interpretation remains speculative because information regarding intramuscular tendon involvement was not consistently reported within the dataset, which precluded robust analysis of its influence on the present findings. Consequently, the inclusion of more detailed diagnostic information in future research is warranted to better contextualize injury trends and to elucidate the underlying physiological mechanisms contributing to observed patterns.

Finally, the 2019–2020 season was truncated due to the coronavirus pandemic (COVID‐19), so may not be a true representation of a full season as clubs were forced to end their season in March 2020, with a small number of clubs resuming and completing their season in July 2020. Additionally, while the 2020–2021 season is a full representation of a domestic season, the impact of COVID‐19 and the associated restrictions may have had a continued influence on the findings for this year, so it should be interpreted with caution.

## 6 Perspectives

This study analyzed 12 seasons of English men's professional domestic football, representing the largest surveillance dataset in this cohort. It provides a contemporary overview of injury incidence rate, burden, and severity, offering valuable benchmarks for practitioners to compare with team‐level data in applied settings.

Future research should move beyond descriptive epidemiology toward more analytical and mechanistic approaches, addressing the limited availability of comprehensive longitudinal data. Greater diagnostic specificity is needed, particularly to explore temporal trends and subtype‐driven injury patterns, which remain underexamined. Methodological advances should include comparisons across league tiers to assess how competitive demands and playing styles influence injury risk, as well as sex‐based analyses to understand differences between men's and women's football.

There is also a need to investigate injury causation more directly through contextual variables, such as activity at the time of injury, supported by systematic video analysis. Integrating measures of physical load and fixture density would further contextualize injury risk. Finally, longitudinal, anonymized player‐level data would enable more precise tracking of exposure, injury history, and recovery, supporting advanced modeling and more effective, targeted prevention strategies.

## Author Contributions

C.C., I.V., and B.S. conceptualized the study. Material preparation, data collection, and curation were performed by all authors. Coaches and medical support staff employed by each club collected the data and transferred it to the research team and current authors. C.C. provided oversight of staff to ensure data were collected appropriately for the injury and illness surveillance programme. All authors were involved in the preparation of the material used for the data collection, and B.S. and I.V. conducted the analysis. B.S. drafted the manuscript. All authors revised the manuscript, contributed to the content, and approved the final version. I.V. is the guarantor.

## Funding

Funding for the current research project was provided by the English Football Association (grant number: 1750119).

## Ethics Statement

This study involves human participants, and ethical approval was granted by the Nottingham Trent University Non‐Invasive Ethical Review Committee to retrospectively analyze FA‐owned data (application number: 116 V2).

## Consent

Informed consent was obtained from all clubs prior to data collection.

## Conflicts of Interest

C.C. is an employee of the English Football Association (The FA). S.B. is an employee of the English Football League (EFL) and a current EFL club. I.V. has received funding as principal investigator from the English Football Association as an Injury Surveillance consultant. No other conflicts of interest to report.

## Supporting information


**Figure S1:** Number of teams that provided both injury and exposure data for matches and training in each season. The dotted vertical line indicates the mandatory sharing of injury data with the Injury and Illness Surveillance Programme (IISP) for EFL Teams (21–22 season).

**Table S1:** Sample characteristics presented as the average (95% confidence interval) per league per season.

**Table S2:** Sample Characteristics with the number of match and training injuries, and associated days absent, adapted for teams providing exposure data.

**Table S3:** Frequency and total severity adapted for teams providing exposure data with corresponding injury incidence rate and burden data for the types of injury that occurred overall, in matches and training.

**Table S4:** Frequency adapted for teams providing exposure data with corresponding injury incidence rate data for the location and diagnoses of injury that occurred overall, in matches and training.

**Table S5:** Frequency and total severity adapted for teams providing exposure data with injury burden data for the location and diagnoses of injury that occurred overall, in matches and training.

**Table S6:** Frequency adapted for teams providing exposure data with corresponding injury incidence rate data for the severity, cause and onset of injury overall, in matches and training.

**Table S7:** Outputs from sensitivity analyses restricted to teams with repeated participation in ≥ 2 seasons, ≥ 3 seasons, and ≥ 4 seasons.

## Data Availability

The data are not publicly available due to their containing information that could compromise the confidentiality of clubs and organizations involved.
