# Supplementary material for: The Football Association Injury and Illness Surveillance Programme: The Incidence, Burden, and Severity of Injuries in English Men's Domestic Football
Source: Scand J Med Sci Sports. 2026 Oct 3;36(10):e70378. doi: 10.1111/sms.70378 (PMC13633597; doi:10.1111/sms.70378)
Supplement: Supplementary file 1 [file SMS-36-e70378-s001.docx]

**Supplementary Table 5.** Frequency and total severity adapted for teams providing exposure data with injury burden data for the location and diagnoses of injury that occurred overall, in matches and training.

| **Injury**  **Location**  *Diagnosis* | **Frequency**  **(n)** | | | **Total Severity**  **(Days Absent)** | | | **Burden**  **[Days Absent/1000h (95% CI)]** | | |  |
| --- | --- | --- | --- | --- | --- | --- | --- | --- | --- | --- |
|  | All | Match | Training | All | Match | Training | All | Match | Training | IBR  (95% CI) |
| **Abdomen** | 148 | 84 | 64 | 3,725 | 1,863 | 1,862 | 1.63  (1.00-2.44) | 5.20  (3.14-7.75) | 0.97  (0.58-1.44) | 5.38  (2.85-10.30) * |
| **Achilles Tendon** | 227 | 120 | 107 | 14,191 | 9,077 | 5,114 | 6.21  (3.76-9.14) | 25.32  (15.38-37.90) | 2.65  (1.61-3.94) | 9.54  (5.14-18.06) * |
| **Ankle** | 1,971 | 1,201 | 770 | 59,356 | 38,072 | 21,284 | 25.96  (15.86-38.37) | 106.19  (65.73-157.22) | 11.04  (6.74-16.36) | 9.62  (5.17-18.01) * |
| *Lateral Ankle Ligament* | 705 | 421 | 284 | 17,553 | 10,496 | 7,057 | 7.68  (4.67-11.34) | 29.28  (17.80-43.38) | 3.66  (2.22-5.44) | 7.97  (4.18-15.37) * |
| *Deltoid Ankle Ligament* | 203 | 133 | 70 | 6,544 | 4,960 | 1,584 | 2.86  (1.74-4.23) | 13.83  (8.52-20.51) | 0.82  (0.50-1.23) | 16.83  (8.91-31.97) * |
| **Arm** | 32 | 18 | 14 | 1,412 | 781 | 631 | 0.62  (0.38-0.92) | 2.18  (1.32-3.25) | 0.33  (0.20-0.49) | 6.65  (3.50-12.81) * |
| **Foot/Toe** | 660 | 437 | 223 | 20,341 | 13,736 | 6,605 | 8.9  (5.40-13.29) | 38.31  (23.42-56.51) | 3.43  (2.10-5.11) | 11.18  (5.93-21.38) * |
| **Head/Face/Neck/Cervical Spine** | 610 | 473 | 137 | 8,753 | 6,518 | 2,235 | 3.83  (2.34-5.69) | 18.18  (11.05-26.96) | 1.16  (0.72-1.72) | 15.68  (8.29-29.96) * |
| *Concussion* | 420 | 330 | 90 | 5,384 | 4,213 | 1,171 | 2.36  (1.44-3.51) | 11.75  (7.14-17.41) | 0.61  (0.37-0.90) | 19.34  (10.21-36.95) * |
| *Head/Face/Neck Injury* | 185 | 141 | 44 | 3,125 | 2,253 | 872 | 1.37  (0.82-2.01) | 6.28  (3.78-9.32) | 0.45  (0.27-0.67) | 13.89  (7.40-26.18) * |
| **Hip/Groin** | 910 | 443 | 467 | 19,457 | 8,886 | 10,571 | 8.51  (5.23-12.72) | 24.78  (15.03-36.63) | 5.48  (3.32-8.19) | 4.52  (2.43-8.68) * |
| *Hip/Groin Muscle/Tendon* | 567 | 267 | 300 | 10,745 | 4,670 | 6,075 | 4.70  (2.48-6.98) | 13.03  (7.96-19.43) | 3.15  (1.93-4.69) | 4.13  (2.20-7.87) * |
| **Knee** | 2,014 | 1,194 | 820 | 114,824 | 80,536 | 34,288 | 50.23  (30.68-74.83) | 224.63  (136.46-330.87) | 17.79  (10.84-26.34) | 12.63  (6.65-23.96) * |
| *Anterior Cruciate (ACL)* | 174 | 142 | 32 | 41,718 | 35,064 | 6,654 | 18.16  (11.17-27.17) | 97.80  (59.64-143.76) | 3.45  (2.09-5.09) | 28.33  (15.12-53.22) * |
| *Medial Collateral (MCL)* | 462 | 299 | 163 | 17,884 | 12,960 | 4,924 | 7.82  (4.77-11.56) | 36.15  (21.99-53.49) | 2.55  (1.56-3.77) | 14.15  (7.52-26.67) * |
| *Meniscal/Cartilage* | 382 | 189 | 193 | 22,804 | 11,864 | 10,940 | 9.98  (6.05-14.71) | 33.09  (20.46-48.92) | 5.68  (3.44-8.46) | 5.83  (3.09-11.13) * |
| **Low Back** | 427 | 170 | 257 | 9,556 | 3,643 | 5,913 | 4.18  (2.53-6.21) | 10.16  (6.19-14.99) | 3.07  (1.88-4.48) | 3.31  (1.74-6.32) * |
| **Lower Leg** | 1,660 | 939 | 721 | 44,374 | 26,384 | 17,990 | 19.41  (11.90-28.72) | 73.59  (45.06-109.63) | 9.33  (5.67-13.92) | 7.88  (4.21-14.87) * |
| *Triceps Surae* | 1,310 | 708 | 602 | 32,991 | 18,332 | 14,659 | 14.43  (8.92-21.42) | 51.13  (31.47-75.58) | 7.61  (4.63-11.28) | 6.72  (3.59-12.63) * |
| **Pelvis/Sacrum** | 259 | 127 | 132 | 3,916 | 1,555 | 2,361 | 1.71  (1.04-2.55) | 4.34  (2.68-6.44) | 1.22  (0.74-1.81) | 3.54  (1.88-6.71) * |
| **Shoulder/Clavicle** | 313 | 228 | 85 | 11,210 | 7,718 | 3,492 | 4.90  (2.99-7.30) | 21.53  (13.11-31.96) | 1.81  (1.08-2.67) | 11.88  (6.34-22.32) * |
| **Sternum/Ribs/Upper Back** | 105 | 68 | 37 | 1,663 | 1,255 | 408 | 0.73  (0.45-1.09) | 3.50  (2.13-5.23) | 0.21  (0.13-0.32) | 16.54  (8.68-31.30) * |
| **Thigh** | 5,434 | 3,234 | 2,200 | 146,286 | 93,071 | 53,215 | 63.99  (38.67-94.83) | 259.59  (159.74-383.81) | 27.61  (16.87-41.12) | 9.40  (5.02-17.91) * |
| *Hamstring Muscle/Tendon* | 3,031 | 2,001 | 1,030 | 92,277 | 64,825 | 27,452 | 40.37  (24.80-59.72) | 180.81  (110.58-269.53) | 14.24  (8.74-21.06) | 12.70  (6.70-24.17) * |
| *Quadriceps Muscle/Tendon* | 1,069 | 454 | 615 | 30,748 | 14,296 | 16,452 | 13.45  (8.14-19.92) | 39.87  (24.60-59.60) | 8.54  (5.22-12.79) | 4.67  (2.48-8.91) * |
| *Adductor Muscle/Tendon* | 1,004 | 529 | 475 | 19,383 | 11,144 | 8,239 | 8.48  (5.15-12.55) | 31.08  (18.96-45.93) | 4.27  (2.64-6.32) | 7.27  (3.87-13.93) * |
| **Wrist/Hand/Finger/Thumb** | 195 | 91 | 104 | 5,985 | 2424 | 3,561 | 2.62  (1.61-3.88) | 6.76  (4.13-10.07) | 1.85  (1.14-2.73) | 3.66  (1.93-6.94) * |
| **Unspecified** | 46 | 23 | 23 | 1,580 | 1,004 | 576 | 0.69  (0.42-1.02) | 2.80  (1.69-4.15) | 0.30  (0.18-0.44) | N/A |

Note: Where corresponding exposure data were unavailable, injuries were excluded from incidence rate and burden calculations. This accounted for 1% and 13% of all injuries for matches and training, respectively, as well as 0.7% and 15% of total days absent from match and training injuries, respectively. Excluding these cases ensured accurate calculations of injury incidence rate and burden (per 1,000 hours). Frequency of injuries (n) and total severity (days absent) represent the adapted total values that were reported alongside corresponding exposure. Lower limb muscle/tendon categories exclude haematoma/contusion/bruise injuries. *greater injury burden in matches compared to training, determined by injury burden ratios (IBR), where effect estimates with associated 95% CIs excluding 1.00 indicate statistical significance. Injury burden ratio was not calculated for unspecified injuries (N/A).
