# Supplementary material for: The Football Association Injury and Illness Surveillance Programme: The Incidence, Burden, and Severity of Injuries in English Men's Domestic Football
Source: Scand J Med Sci Sports. 2026 Oct 3;36(10):e70378. doi: 10.1111/sms.70378 (PMC13633597; doi:10.1111/sms.70378)
Supplement: Supplementary file 2 [file SMS-36-e70378-s002.docx]

**Supplementary Table 6.** Frequency adapted for teams providing exposure data with corresponding injury incidence rate data for the severity, cause and onset of injury overall, in matches and training.

|  | **All** | | **Match** | | | **Training** | |  |
| --- | --- | --- | --- | --- | --- | --- | --- | --- |
|  | **No. of Injuries**  **(n)** | **Incidence Rate**  **[Injuries/1000h (95% CI)]** | **Match**  **No. of Injuries (n)** | **Match Incidence Rate [Injuries/1000h (95% CI)]** | **Training**  **No. of Injuries (n)** | | **Training Incidence Rate [Injuries/1000h (95% CI)]** | **IRR**  **(95% CI)** |
| **Severity** | **(n=14,954)** |  | **(n=8,816)** |  | **(n=6,138)** | |  |  |
| Minor | 4,131 | 1.81  (1.10-2.68) | 2,200 | 6.14  (3.68-9.10) | 1,931 | | 1.00  (0.61-1.50) | 6.13  (3.24-11.71) * |
| Moderate | 6,077 | 2.66  (1.62-3.95) | 3,602 | 10.05  (6.19-14.91) | 2,475 | | 1.28  (0.78-1.90) | 7.82  (4.17-14.81) * |
| Severe | 3,716 | 1.63  (0.98-2.39) | 2,343 | 6.54  (3.99-9.60) | 1,373 | | 0.71  (0.44-1.07) | 9.17  (4.89-17.25) * |
| Major | 1,030 | 0.45  (0.28-0.67) | 671 | 1.87  (1.12-2.79) | 359 | | 0.19  (0.11-0.28) | 10.05  (5.28-19.32) * |
| **Cause** | **(n=13,547)** |  | **(n=8,173)** |  | **(n=5,374)** | |  |  |
| Contact | 4,488 | 1.96  (1.20-2.90) | 3,290 | 9.18  (5.65-13.65) | 1,198 | | 0.62  (0.38-0.94) | 14.76  (7.76-28.29) * |
| Non-Contact | 8,856 | 3.87  (2.38-5.76) | 4,800 | 13.39  (8.13-19.86) | 4,056 | | 2.10  (1.28-3.15) | 6.36  (3.35-12.06) * |
| Cumulative | 203 | 0.09  (0.05-0.13) | 83 | 0.23  (0.13-0.36) | 120 | | 0.06  (0.04-0.09) | 3.72  (1.85-7.43) * |
| **Onset** | **(n=12,460)** |  | **(n=7,651)** |  | **(n=4,809)** | |  |  |
| Acute | 10,022 | 4.38  (2.68-6.58) | 6,430 | 17.93  (11.08-26.35) | 3,592 | | 1.86  (1.14-2.77) | 9.62  (5.10-18.38) * |
| Gradual | 2,438 | 1.07  (0.66-1.60) | 1,221 | 3.41  (2.09-5.05) | 1,217 | | 0.63  (0.38-0.94) | 5.39  (2.82-10.18) * |

Note: Frequency of injuries (n) represents all injuries with a known severity, onset and cause of injury. Therefore, where data was unspecified, the injuries were removed from the total number of injuries. Additionally, where corresponding exposure data were unavailable, injuries were excluded from incidence rate calculations. This accounted for 1% and 13% of all injuries in matches and training, respectively. Excluding these cases ensured accurate calculations of injury incidence rate (per 1,000 hours). *greater injury incidence rate in matches compared to training; determined by injury incidence rate ratios (IRR), where effect estimates with associated 95% CIs excluding 1.00 indicate statistical significance.
