# Supplementary material for: The Football Association Injury and Illness Surveillance Programme: The Incidence, Burden, and Severity of Injuries in English Men's Domestic Football
Source: Scand J Med Sci Sports. 2026 Oct 3;36(10):e70378. doi: 10.1111/sms.70378 (PMC13633597; doi:10.1111/sms.70378)
Supplement: Supplementary file 3 [file SMS-36-e70378-s003.docx]

**Supplementary Figure 1.** Number of teams that provided both injury and exposure data for matches and training in each season. The dotted vertical line indicates the mandatory sharing of injury data with the Injury and Illness Surveillance Programme (IISP) for EFL Teams (21-22 season).
