# Supplementary material for: The Football Association Injury and Illness Surveillance Programme: The Incidence, Burden, and Severity of Injuries in English Men's Domestic Football
Source: Scand J Med Sci Sports. 2026 Oct 3;36(10):e70378. doi: 10.1111/sms.70378 (PMC13633597; doi:10.1111/sms.70378)
Supplement: Supplementary file 4 [file SMS-36-e70378-s004.docx]

|  | **Season** | | | | | | | | | | | | |
| --- | --- | --- | --- | --- | --- | --- | --- | --- | --- | --- | --- | --- | --- |
|  | **13-14** | **14-15** | **15-16** | **16-17** | **17-18** | **18-19** | **19-20** | **20-21** | **21-22** | **22-23** | **23-24** | **24-25** | **Total** |
| Total Teams (n) | 13 | 18 | 13 | 19 | 15 | 18 | 22 | 15 | 70 | 71 | 70 | 72 | 416 |
| Teams Providing Match  Exposure (n) | 13 | 16 | 12 | 19 | 14 | 17 | 22 | 15 | 69 | 71 | 70 | 72 | 410 |
| Match Exposure (h) | 10,231.5 | 13,205.2 | 9,828.2 | 14,789.2 | 11,365.5 | 14,001.2 | 17,687.4 | 13,117.1 | 62,058.9 | 63,315.4 | 64,621.9 | 64,306.3 | 358,527.8 |
| Match Injuries (n) | 267 | 293 | 192 | 396 | 264 | 374 | 440 | 283 | 1,630 | 1,617 | 1,542 | 1,552 | 8,850 |
| Days Absent Due to Match Injuries (Days) | 7,161 | 9,737 | 6,498 | 10,303 | 6,505 | 7,875 | 12,663 | 8,289 | 51,272 | 61,691 | 53,952 | 60,577 | 296,523 |
| Teams Providing Training Exposure (n) | 9 | 14 | 10 | 19 | 14 | 18 | 20 | 13 | 65 | 63 | 64 | 59 | 368 |
| Training Exposure (h) | 41,008.8 | 61,891.5 | 48,436.5 | 85,181.1 | 65,378.1 | 97,781.4 | 110,358.4 | 60,851.4 | 318,325.8 | 298,657.3 | 384,596.6 | 355,034.8 | 1,927,501.8 |
| Training Injuries (n) | 245 | 255 | 154 | 389 | 272 | 370 | 311 | 247 | 1,029 | 1,050 | 975 | 864 | 6,161 |
| Days Absent Due to Training Injuries (Days) | 4,114 | 6,051 | 3,442 | 8,770 | 6,577 | 7,524 | 7,484 | 5,900 | 28,122 | 33,970 | 29,994 | 28,158 | 170,106 |

**Supplementary Table 2.** Sample Characteristics with the number of match and training injuries, and associated days absent, adapted for teams providing exposure data.

Note: n = frequency; h = hours. The 21-22 season represented the point at which mandatory injury data sharing with the Injury and Illness Surveillance Programme (IISP) was implemented across EFL teams. Where corresponding exposure data were unavailable, injuries were excluded from incidence rate and burden calculations. This accounted for 1% and 13% of all injuries for matches and training, respectively, as well as 0.7% and 15% of total days absent from match and training injuries, respectively. Excluding these cases ensured accurate calculations of injury incidence rate and burden (per 1,000 hours).
