# Supplementary material for: The Football Association Injury and Illness Surveillance Programme: The Incidence, Burden, and Severity of Injuries in English Men's Domestic Football
Source: Scand J Med Sci Sports. 2026 Oct 3;36(10):e70378. doi: 10.1111/sms.70378 (PMC13633597; doi:10.1111/sms.70378)
Supplement: Supplementary file 5 [file SMS-36-e70378-s005.docx]

| **Injury**  **Location**  *Diagnosis* | **Frequency**  **(n)** | | | **Incidence Rate**  **[Injuries/1000h (95% CI)]** | | |  |
| --- | --- | --- | --- | --- | --- | --- | --- |
|  | All | Match | Training | All | Match | Training | IRR  (95% CI) |
| **Abdomen** | 148 | 84 | 64 | 0.06  (0.04-0.10) | 0.23  (0.13-0.36) | 0.03  (0.02-0.05) | 7.06  (3.41-14.92) * |
| **Achilles Tendon** | 227 | 120 | 107 | 0.10  (0.06-0.15) | 0.33  (0.20-0.51) | 0.06  (0.03-0.09) | 6.03  (3.05-11.74) * |
| **Ankle** | 1,971 | 1,201 | 770 | 0.86  (0.53-1.29) | 3.35  (2.04-5.00) | 0.40  (0.24-0.59) | 8.39  (4.40-16.06) * |
| *Lateral Ankle Ligament* | 705 | 421 | 284 | 0.31  (0.19-0.46) | 1.17  (0.71-1.76) | 0.15  (0.09-0.22) | 7.97  (4.18-15.37) * |
| *Deltoid Ankle Ligament* | 203 | 133 | 70 | 0.09  (0.05-0.13) | 0.37  (0.21-0.57) | 0.04  (0.02-0.06) | 10.21  (5.06-20.89) * |
| **Arm** | 32 | 18 | 14 | 0.01  (0.01-0.02) | 0.05  (0.02-0.09) | 0.01  (0.00-0.01) | 6.91  (2.51-19.35) * |
| **Foot/Toe** | 660 | 437 | 223 | 0.29  (0.18-0.43) | 1.22  (0.73-1.83) | 0.12  (0.07-0.17) | 10.54  (5.49-20.50) * |
| **Head/Face/Neck/Cervical Spine** | 610 | 473 | 137 | 0.27  (0.16-0.40) | 1.32  (0.80-1.97) | 0.07  (0.04-0.11) | 18.56  (9.65-36.76) * |
| *Concussion* | 420 | 330 | 90 | 0.18  (0.11-0.28) | 0.92  (0.56-1.39) | 0.05  (0.03-0.07) | 19.71  (10.16-39.51) * |
| *Head/Face/Neck Injury* | 185 | 141 | 44 | 0.08  (0.05-0.12) | 0.39  (0.23-0.60) | 0.02  (0.01-0.04) | 17.23  (8.50-36.84) * |
| **Hip/Groin** | 910 | 443 | 467 | 0.40  (0.24-0.60) | 1.24  (0.75-1.84) | 0.24  (0.15-0.36) | 5.10  (2.68-9.72) * |
| *Hip/Groin Muscle/Tendon* | 567 | 267 | 300 | 0.25  (0.15-0.37) | 0.74  (0.45-1.12) | 0.16  (0.09-0.23) | 4.78  (2.49-9.38) * |
| **Knee** | 2,014 | 1,194 | 820 | 0.88  (0.54-1.31) | 3.33  (2.04-4.96) | 0.43  (0.26-0.64) | 7.83  (4.15-15.06) * |
| *Anterior Cruciate (ACL)* | 174 | 142 | 32 | 0.08  (0.05-0.12) | 0.40  (0.23-0.60) | 0.02  (0.01-0.03) | 23.86  (11.47-52.74) * |
| *Medial Collateral (MCL)* | 462 | 299 | 163 | 0.20  (0.12-0.30) | 0.83  (0.50-1.24) | 0.08  (0.05-0.13) | 9.86  (5.13-19.27) * |
| *Meniscal/Cartilage* | 382 | 189 | 193 | 0.17  (0.10-0.25) | 0.53  (0.31-0.79) | 0.10  (0.06-0.15) | 5.26  (2.72-10.28) * |
| **Low Back** | 427 | 170 | 257 | 0.19  (0.11-0.28) | 0.47  (0.28-0.71) | 0.13  (0.08-0.20) | 3.56  (1.83-7.04) * |
| **Lower Leg** | 1,660 | 939 | 721 | 0.73  (0.44-1.08) | 2.62  (1.58-3.90) | 0.37  (0.23-0.56) | 7.00  (3.67-13.51) * |
| *Triceps Surae* | 1,310 | 708 | 602 | 0.57  (0.35-0.85) | 1.97  (1.19-2.94) | 0.31  (0.19-0.46) | 6.32  (3.34-12.21) * |
| **Pelvis/Sacrum** | 259 | 127 | 132 | 0.11  (0.07-0.17) | 0.35  (0.21-0.54) | 0.07  (0.04-0.10) | 5.17  (2.63-10.24) * |
| **Shoulder/Clavicle** | 313 | 228 | 85 | 0.14  (0.08-0.21) | 0.64  (0.38-0.95) | 0.04  (0.02-0.07) | 14.42  (7.34-28.74) * |
| **Sternum/Ribs/Upper Back** | 105 | 68 | 37 | 0.05  (0.03-0.07) | 0.19  (0.11-0.29) | 0.02  (0.01-0.03) | 9.88  (4.69-21.84) * |
| **Thigh** | 5,434 | 3,234 | 2,200 | 2.38  (1.44-3.51) | 9.02  (5.50-13.52) | 1.14  (0.69-1.71) | 7.90  (4.15-15.02) * |
| *Hamstring Muscle/Tendon* | 3,031 | 2,001 | 1,030 | 1.33  (0.82-1.96) | 5.58  (3.40-8.36) | 0.53  (0.32-0.80) | 10.44  (5.55-19.74) * |
| *Quadriceps Muscle/Tendon* | 1,069 | 454 | 615 | 0.47  (0.29-0.70) | 1.27  (0.76-1.89) | 0.32  (0.19-0.47) | 3.97  (2.08-7.60) * |
| *Adductor Muscle/Tendon* | 1,004 | 529 | 475 | 0.44  (0.27-0.66) | 1.48  (0.88-2.20) | 0.25  (0.15-0.37) | 5.99  (3.12-11.32) * |
| **Wrist/Hand/Finger/Thumb** | 195 | 91 | 104 | 0.09  (0.05-0.13) | 0.25  (0.15-0.39) | 0.05  (0.03-0.08) | 4.70  (2.30-9.59) * |
| **Unspecified** | 46 | 23 | 23 | 0.02  (0.01-0.03) | 0.06  (0.03-0.11) | 0.01  (0.01-0.02) | N/A |

**Supplementary Table 4.** Frequency adapted for teams providing exposure data with corresponding injury incidence rate data for the location and diagnoses of injury that occurred overall, in matches and training.

Note: Where corresponding exposure data were unavailable, injuries were excluded from incidence rate calculations. This accounted for 1% and 13% of all injuries for matches and training, respectively. Excluding these cases ensured accurate calculations of injury incidence rate (per 1,000 hours). Frequency of injuries (n) represents the adapted total values that were reported alongside corresponding exposure. Lower limb muscle/tendon categories exclude haematoma/contusion/bruise injuries. *greater injury incidence rate in matches compared to training; determined via injury incidence rate ratios (IRR), where effect estimates with associated 95% CIs excluding 1.00 indicate statistical significance. Injury incidence rate ratio was not calculated for unspecified injuries (N/A).
