# Supplementary material for: The Football Association Injury and Illness Surveillance Programme: The Incidence, Burden, and Severity of Injuries in English Men's Domestic Football
Source: Scand J Med Sci Sports. 2026 Oct 3;36(10):e70378. doi: 10.1111/sms.70378 (PMC13633597; doi:10.1111/sms.70378)
Supplement: Supplementary file 6 [file SMS-36-e70378-s006.docx]

| **Injury Type** | **Frequency**  **(n)** | | | **Incidence Rate**  **[Injuries/1000h (95% CI)]** | | |  | **Total Severity**  **[Days Absent]** | | | | **Burden**  **[Days Absent/1000h (95% CI)]** | | | |  | |
| --- | --- | --- | --- | --- | --- | --- | --- | --- | --- | --- | --- | --- | --- | --- | --- | --- | --- |
|  | All | Match | Training | All | Match | Training | IRR  (95% CI) | All | Match | Training | All | | Match | Training | IBR  (95% CI) | |  |
| **Muscle Strain/Tear/Rupture/Cramps** | 6,689 | 3,734 | 2,955 | 2.93  (1.77-4.35) | 10.41  (6.46-15.44) | 1.53  (0.94-2.28) | 6.79  (3.57-13.02) * | 162,082 | 97,348 | 64,734 | 70.90  (43.45-104.73) | | 271.52  (165.73-405.45) | 33.58  (20.44-49.85) | 8.08  (4.26-15.35) ** | |  |
| **Ligament Sprain/Tear/Rupture** | 2,732 | 1,773 | 959 | 1.20  (0.73-1.76) | 4.95  (2.98-7.31) | 0.50  (0.30-0.74) | 9.94  (5.30-18.75) * | 126,267 | 93,088 | 33,179 | 55.23  (33.75-81.33) | | 259.4  (159.47-386.62) | 17.21  (10.43-25.46) | 15.08  (8.09-28.70) ** | |  |
| **Tendon Injury/Rupture/Tendinosis** | 1,248 | 679 | 569 | 0.55  (0.33-0.82) | 1.89  (1.15-2.80) | 0.30  (0.18-0.44) | 6.42  (3.37-12.22) * | 57,034 | 35,061 | 21,973 | 24.95  (15.17-37.21) | | 97.79  (59.13-144.83) | 11.40  (6.91-16.93) | 8.58  (4.55-16.39) ** | |  |
| **Haematoma/Contusion/Bruise** | 1,064 | 766 | 298 | 0.47  (0.28-0.69) | 2.14  (1.29-3.19) | 0.15  (0.09-0.23) | 13.82  (7.35-26.51) * | 10,610 | 7,725 | 2,885 | 4.64  (2.82-6.83) | | 21.55  (13.22-32.28) | 1.50  (0.91-2.20) | 14.40  (7.64-27.55) ** | |  |
| **Concussion** | 420 | 330 | 90 | 0.18  (0.11-0.28) | 0.92  (0.56-1.39) | 0.05  (0.03-0.07) | 19.71  (10.16-39.51) * | 5,384 | 4,213 | 1,171 | 2.36  (1.44-3.51) | | 11.75  (7.14-17.41) | 0.61  (0.37-0.90) | 19.34  (10.21-36.95) ** | |  |
| **Bursitis/Impingement/Synovitis** | 389 | 191 | 198 | 0.17  (0.10-0.26) | 0.53  (0.31-0.81) | 0.10  (0.06-0.21) | 5.19  (2.66-10.28) * | 6,680 | 3,766 | 2,914 | 2.92  (1.78-4.35) | | 10.50  (6.40-15.56) | 1.51  (0.93-2.24) | 6.95  (3.68-13.31) ** | |  |
| **Cartilage/Disc/Meniscus** | 551 | 256 | 295 | 0.24  (0.15-0.36) | 0.71  (0.42-1.08) | 0.15  (0.09-0.23) | 4.67  (2.39-9.05) * | 29,496 | 14,439 | 15,057 | 12.40  (7.55-18.40) | | 40.27  (24.58-59.79) | 7.81  (4.68-11.53) | 5.16  (2.74-9.79) ** | |  |
| **Bone Fracture** | 623 | 410 | 213 | 0.27  (0.17-0.41) | 1.14  (0.69-1.71) | 0.11  (0.07-0.16) | 10.35  (5.40-20.01) * | 34,492 | 22,239 | 12,253 | 15.09  (9.14-22.44) | | 62.03  (38.08-90.98) | 6.36  (3.86-9.39) | 9.76  (5.14-18.62) ** | |  |
| **Pain (Undiagnosed)** | 337 | 156 | 181 | 0.15  (0.09-0.22) | 0.44  (0.26-0.66) | 0.09  (0.06-0.14) | 4.63  (2.38-9.18) * | 5,189 | 2,245 | 2,944 | 2.27  (1.38-3.36) | | 6.26  (3.81-9.37) | 1.53  (0.94-2.28) | 4.10  (3.17-7.83) ** | |  |
| **Other** | 915 | 531 | 384 | 0.40  (0.24-0.59) | 1.48  (0.90-2.21) | 0.20  (0.12-0.30) | 7.43  (3.92-14.36) * | 28,349 | 15,556 | 12,793 | 12.40  (7.55-18.40) | | 43.39  (26.25-64.00) | 6.64  (4.06-9.82) | 6.54  (3.47-12.47) ** | |  |
| **Unspecified** | 43 | 24 | 19 | 0.02  (0.01-0.03) | 0.07  (0.03-0.11) | 0.01  (0.00-0.02) | N/A | 1,046 | 843 | 203 | 0.46  (0.28-0.68) | | 2.35  (1.43-3.51) | 0.11  (0.06-0.16) | N/A | |  |

**Supplementary Table 3.** Frequency and total severity adapted for teams providing exposure data with corresponding injury incidence rate and burden data for the types of injury that occurred overall, in matches and training.

Note: Where corresponding exposure data were unavailable, injuries were excluded from incidence rate and burden calculations. This accounted for 1% and 13% of all injuries for matches and training, respectively, as well as 0.7% and 15% of total days absent from match and training injuries, respectively. Excluding these cases ensured accurate calculations of injury incidence rate and burden (per 1,000 hours). Frequency of injuries (n) and total severity (days absent) represent the adapted total values that were reported alongside corresponding exposure. *greater injury incidence rate in matches compared to training, **greater injury burden in matches compared to training; determined via injury incidence rate ratios (IRR) and injury burden ratios (IBR), where effect estimates with associated 95% CIs excluding 1.00 indicate statistical significance. Injury incidence rate and burden ratios were not calculated for unspecified injuries (N/A).
