# Supplementary material for: The Football Association Injury and Illness Surveillance Programme: The Incidence, Burden, and Severity of Injuries in English Men's Domestic Football
Source: Scand J Med Sci Sports. 2026 Oct 3;36(10):e70378. doi: 10.1111/sms.70378 (PMC13633597; doi:10.1111/sms.70378)
Supplement: Supplementary file 7 [file SMS-36-e70378-s007.docx]

| **Repeated Team Participation**  **(Seasons)** | **Frequency (N)** | **Total Severity (Days Absent)** | **Injury Incidence Rate (95% CI)** | **% Change** | ***P* Value** | **Injury Burden (95% CI)** | **% Change** | ***P* Value** |
| --- | --- | --- | --- | --- | --- | --- | --- | --- |
| **Match Injuries** |  |  |  |  |  |  |  |  |
| ≥ 2 | 8,756 | 293,178 | 24.77 (23.84-25.66) | 0 | 0.992 | 831.58 (798.76-864.29) | 5 | <0.001* |
| ≥ 3 | 8,528 | 286,812 | 24.96 (24.02-25.90) | -0.2 | 0.777 | 842.43 (809.80-875.54) | 5 | <0.001* |
| ≥ 4 | 7,845 | 266,279 | 24.64 (23.70-25.55) | -0.2 | 0.821 | 839.55 (805.21-873.79) | 5 | <0.001* |
| **Training Injuries** |  |  |  |  |  |  |  |  |
| ≥ 2 | 6,089 | 168,695 | 3.56 (3.32-3.79) | -4 | <0.001* | 104.32 (97.14-111.14) | 1 | 0.355 |
| ≥ 3 | 5,784 | 159,714 | 3.63 (3.38-3.88) | -4 | <0.001* | 106.07 (98.69-113.37) | 1 | 0.368 |
| ≥ 4 | 5,116 | 145,093 | 3.55 (3.27-3.79) | -3 | <0.001* | 106.53 (98.31-114.17) | 1 | 0.259 |
| **Match Muscle Injuries** |  |  |  |  |  |  |  |  |
| ≥ 2 | 3,693 | 96,397 | 10.51 (9.97-11.03) | 3 | 0.003* | 277.10 (261.30-293.47) | 10 | <0.001* |
| ≥ 3 | 3,593 | 93,765 | 10.58 (10.03-11.13) | 3 | 0.006* | 279.02 (262.50-295.67) | 11 | <0.001* |
| ≥ 4 | 3,326 | 87,058 | 10.50 (9.92-11.08) | 2 | 0.015* | 278.13 (260.80-295.87) | 11 | <0.001* |
| **Training Muscle Injuries** |  |  |  |  |  |  |  |  |
| ≥ 2 | 2,907 | 64,001 | 1.66 (1.54-1.78) | -2 | 0.051 | 38.68 (35.94-41.27) | 4 | 0.001* |
| ≥ 3 | 2,741 | 60,193 | 1.69 (1.55-1.81) | -2 | 0.076 | 38.95 (36.02-41.64) | 4 | 0.002* |
| ≥ 4 | 2,429 | 53,531 | 1.65 (1.51-1.78) | -1 | 0.567 | 38.26 (35.19-41.09) | 5 | 0.001* |
| **Match Ligament Injuries** |  |  |  |  |  |  |  |  |
| ≥ 2 | 1,760 | 92,601 | 4.97 (4.70-5.24) | -0.2 | 0.774 | 259.91 (241.43-277.98) | 2 | 0.120 |
| ≥ 3 | 1,707 | 90,142 | 4.99 (4.71-5.26) | -0.5 | 0.618 | 261.94 (243.07-280.18) | 2 | 0.189 |
| ≥ 4 | 1,569 | 84,536 | 4.92 (4.63-5.21) | -0.1 | 0.878 | 263.60 (243.68-282.15) | 3 | 0.145 |
| **Training Ligament Injuries** |  |  |  |  |  |  |  |  |
| ≥ 2 | 952 | 32,934 | 0.53 (0.48-0.57) | -5 | <0.001* | 20.26 (17.92-22.57) | 2 | 0.290 |
| ≥ 3 | 920 | 32,101 | 0.55 (0.50-0.59) | -5 | <0.001* | 21.31 (18.86-23.76) | 3 | 0.189 |
| ≥ 4 | 797 | 29,341 | 0.52 (0.47-0.56) | -4 | 0.004* | 21.24 (18.60-23.78) | 2 | 0.268 |

**Supplementary Table 7.** Outputs from sensitivity analyses restricted to teams with repeated participation in ≥2 seasons, ≥3 seasons, and ≥4 seasons.

Note: N = frequency. Injury incidence rate is presented as the number of injuries per 1,000h, and injury burden as the number of days absent per 1,000h, with associated 95% confidence intervals (CIs). *indicates a significant temporal trend. Frequency and total severity have been adapted for teams providing exposure data to ensure the accurate calculation of injury incidence rate and burden (per 1,000h). Total teams included per season for match-related information were: ≥2 seasons = 83, ≥3 seasons = 76, ≥4 seasons = 67. Total teams included per season for training-related information were: ≥2 seasons = 80, ≥3 seasons = 68, ≥4 seasons = 56. Corresponding team-seasons for match-related information were: ≥2 seasons = 405, ≥3 seasons = 391, ≥4 seasons = 364. Corresponding team-seasons for training-related information were: ≥2 seasons = 363, ≥3 seasons = 339, ≥4 seasons = 303.
