# Supplementary material for: The Football Association Injury and Illness Surveillance Programme: The Incidence, Burden, and Severity of Injuries in English Men's Domestic Football
Source: Scand J Med Sci Sports. 2026 Oct 3;36(10):e70378. doi: 10.1111/sms.70378 (PMC13633597; doi:10.1111/sms.70378)
Supplement: Supplementary file 8 [file SMS-36-e70378-s008.docx]

**Supplementary Table 1.** Sample characteristics presented as the average (95% confidence interval) per league per season.

|  | **EPL (2016-2021)** | | **Championship (2013-2025)** | | **League One (2016-2025)** | | **League Two (2016-2025)** | |  |
| --- | --- | --- | --- | --- | --- | --- | --- | --- | --- |
|  | **Total** | **Average**  **(95% CI)** | **Total** | **Average**  **(95% CI)** | **Total** | **Average**  **(95% CI)** | **Total** | **Average**  **(95% CI)** | |
| Total Teams (n) | 13 | 3  (1-4) | 193 | 16  (12-20) | 109 | 12  (4-21) | 101 | 11  (2-20) | |
| Teams Providing Match  Exposure (n) | 11 | 2  (1-4) | 190 | 16  (12-20) | 109 | 12  (4-21) | 100 | 11  (2-20) | |
| Match Exposure (h) | 8,119.1 | 1,623.8  (794.6-2,453.0) | 160,743.3 | 13,395.3  (9,664.7-17,125.8) | 101,803.1 | 11,311.5  (3,388.5-19,234.4) | 87,862.4 | 9,762.5  (1,879.9-17645.1) | |
| Match Injuries (n) | 212 | 42  (15-70) | 4,054 | 338  (239-436) | 2,470 | 274  (81-468) | 2,201 | 245  (47-442) | |
| Teams Providing Training Exposure (n) | 12 | 2  (1-4) | 171 | 14  (10-18) | 98 | 11  (3-18) | 87 | 10  (2-17) | |
| Training Exposure (h) | 60,373.4 | 12074.7  (6799.1-17350.3) | 849626.6 | 70,802.2  (51,366.4-90,238.0) | 497053.8 | 55,228.2  (14,886.2-95,570.2) | 520,448.1 | 57,827.6  (10,631.0-105,024.2) | |
| Training Injuries (n) | 244 | 49  (14-84) | 3,542 | 295  (225-366) | 1,777 | 197  (70-325) | 1,486 | 165  (36-294) | |

Note: n = number of teams; h = hours; 95% CI = 95% confidence interval. Data for each sample characteristic is presented as total and average (95% CI) per league per season. For total teams, and total teams providing either match or training exposure, the data represented as totals are reported as the number of team seasons across the study period. For match exposure, match injuries, training exposure and training injuries, this data represents a summed total for the sample characteristics. The number of seasons each league’s data was reported for have been specified in the table headings. The 21-22 season represented the point at which mandatory injury data sharing with the Injury and Illness Surveillance Programme (IISP) was implemented across EFL teams.
